# Supplementary figures and images for: Immunization of cows with HIV envelope trimers generates broadly neutralizing antibodies to the V2-apex from the ultralong CDRH3 repertoire
Source: PLoS Pathog. 2024 Sep 9;20(9):e1012042. doi: 10.1371/journal.ppat.1012042 (PMC11412654; doi:10.1371/journal.ppat.1012042)

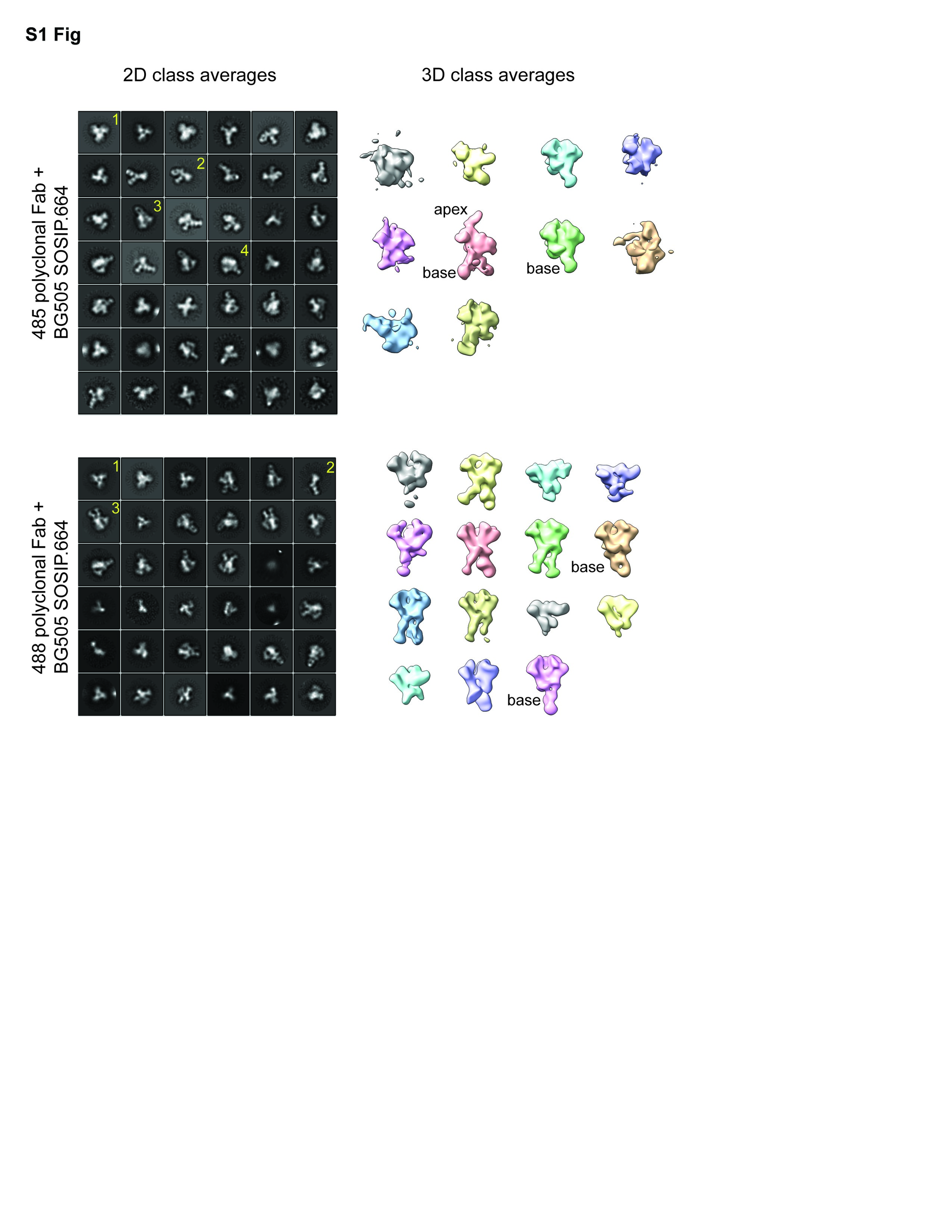

Supplement: S1 Fig — Polyclonal IgG was isolated from cow-485 and cow-488, digested to Fab, incubated with BG505 SOSIP.664, purified by size-exclusion chromatography and imaged by negative-stain EM. 2D and 3D class averages (oriented as side views with viral membrane at the bottom) are shown for each dataset. Annotated observations in the 2D classes are as follow: 1) unbound Env trimer, 2) Fab-bound protomers (dissociated trimer), 3) gp41 base-directed Fabs in complex with trimer, 4) apex-directed Fabs in complex with trimer. Interpretable 3D classes reveal predominantly base-directed (both cows), and apex-directed antibody responses (cow-485). (TIF) [file ppat.1012042.s001.tif]

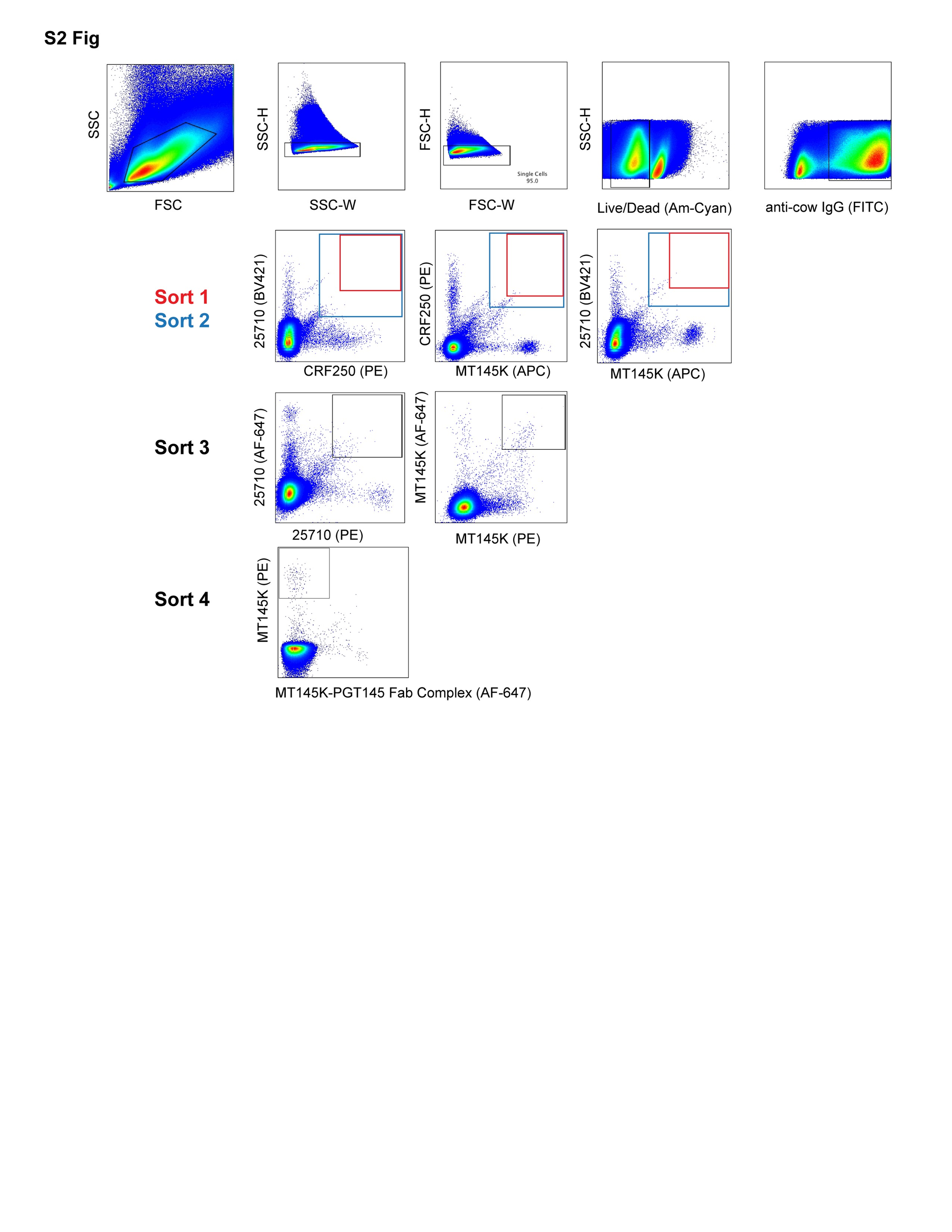

Supplement: S2 Fig — Each sort was completed using samples from both cow-488 and cow-485. The top row shows the overall gating to sort the B cell population and subsequent lines show the strategy for each sort with approximate gating. Sort 1 and Sort 2 isolated cells that had affinity for at least two baits including 25710 SOSIP, CRF250 SOSIP, and MT145K SOSIP. Sort 3 isolated cells that double bound the same bait, either 25710 or MT145K. Sort 4 isolated V2-apex specific cells that bound bait MT145K. (TIF) [file ppat.1012042.s002.tif]

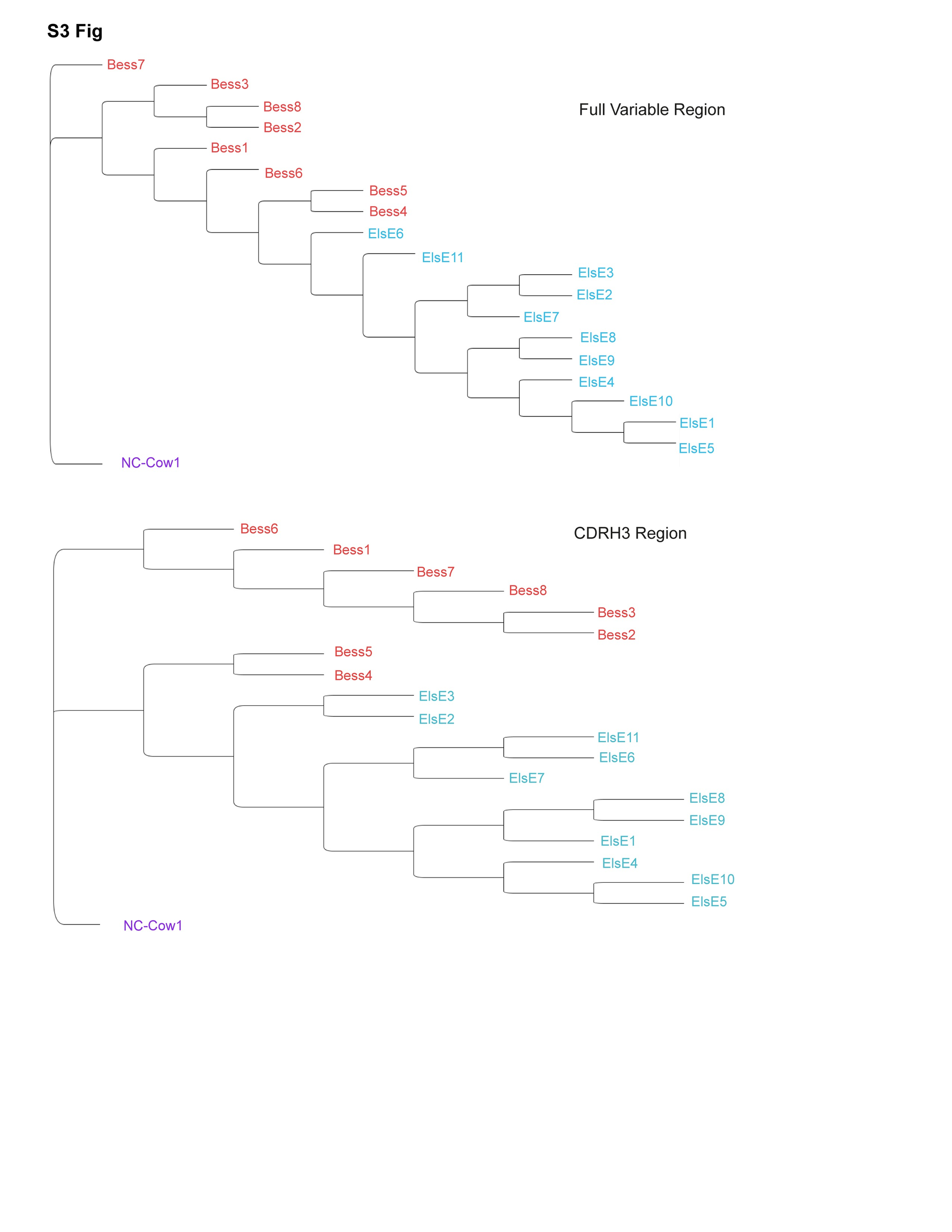

Supplement: S3 Fig — Else, Bess, and NC-Cow1 full variable regions (top) and the CDRH3 regions (bottom) were aligned by amino acid and categorized into a phylogenetic trees based on their amino acid similarity. Bess antibodies are colored in red, ElsE in blue, and NC-Cow1 in purple. (TIF) [file ppat.1012042.s003.tif]

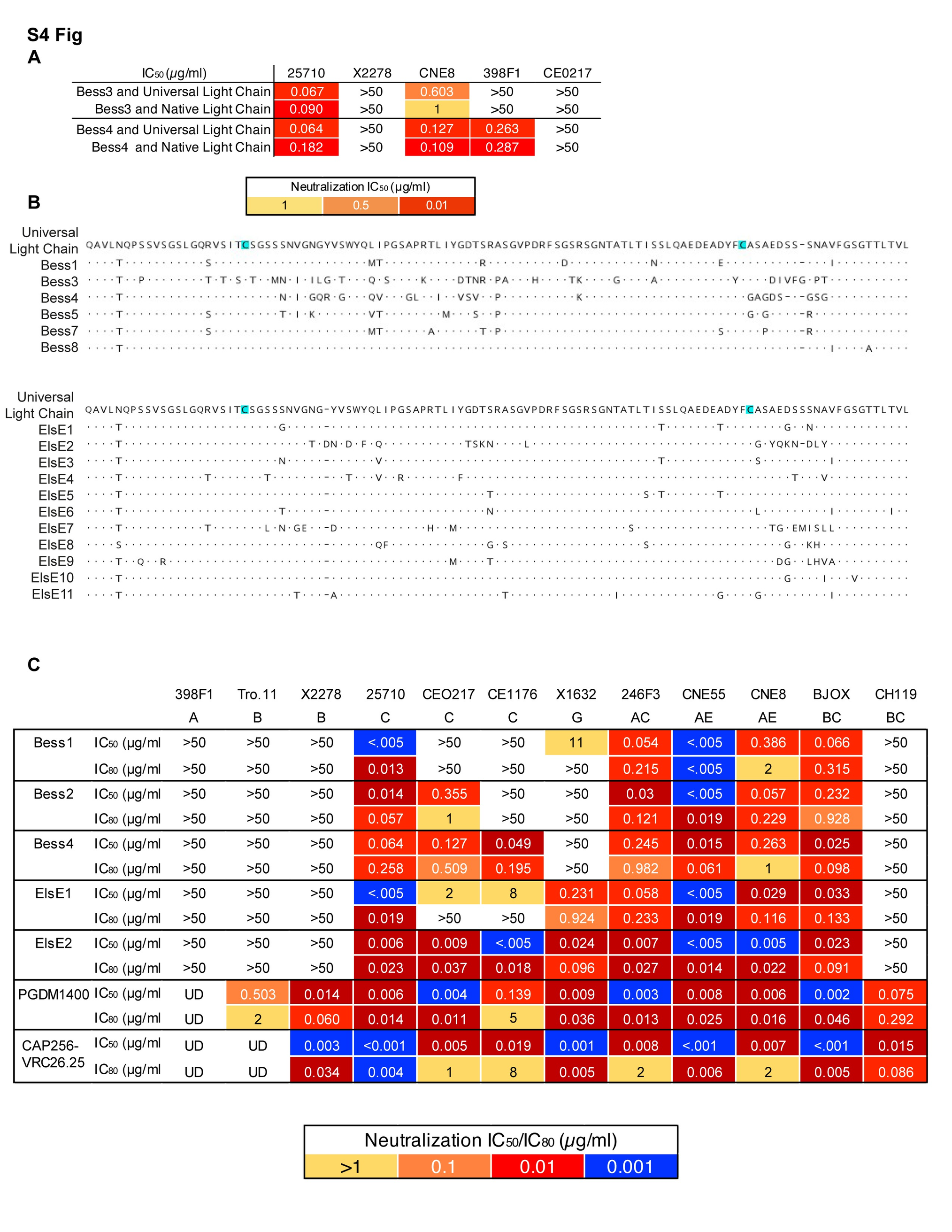

Supplement: S4 Fig — (A) Comparison of Bess3 and Bess4 neutralizing titers when expressed with native or universal light chain. (B) Alignment of native Bess and ElsE Light Chains with cow universal light chain. (C) Comparison of a few representative Bess and ElsE antibodies with PGDM1400 and CAP256-VRC26.25 IC50 (μg/ml) neutralization titers. (TIF) [file ppat.1012042.s004.tif]

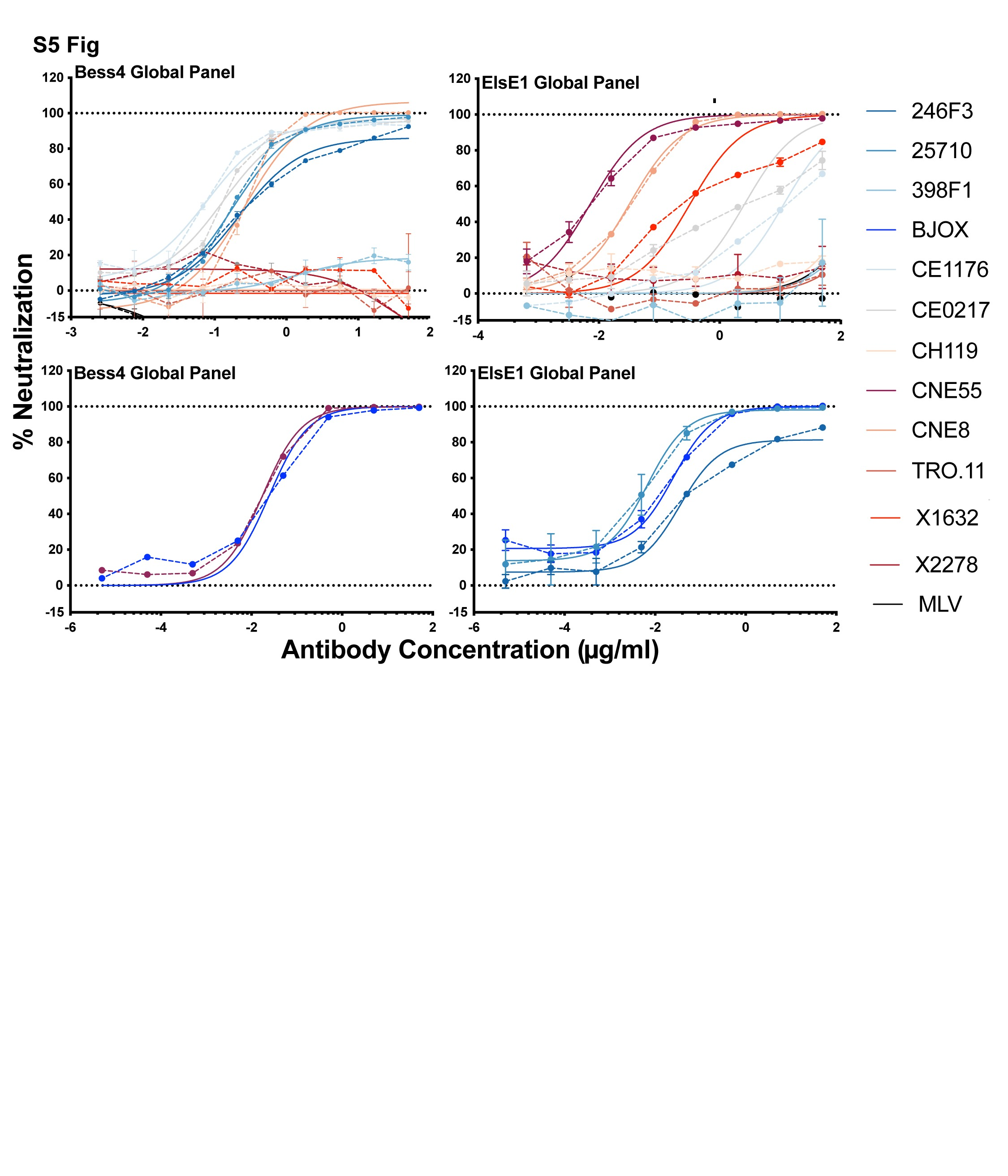

Supplement: S5 Fig — Neutralization curves are shown for the 12-virus global panel for the two bnAbs, Bess4 and ElsE1. Neutralization curves are shown across two different plots depending on the serum dilution factor used in the assay. (TIF) [file ppat.1012042.s005.tif]

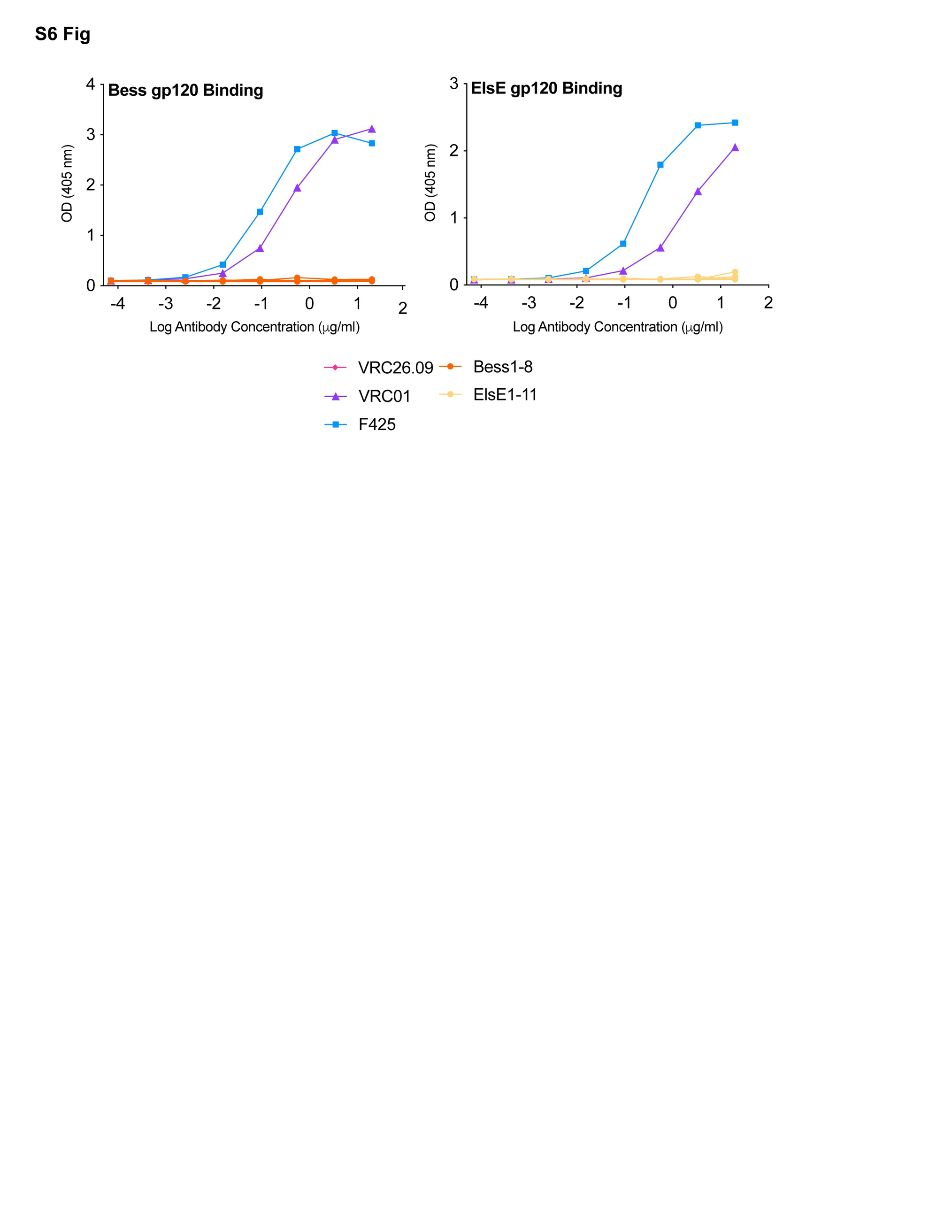

Supplement: S6 Fig — All Bess and ElsE antibodies were measured for binding to BG505 gp120 using ELISA. Antibody controls include positive controls VRC01 and F425, and negative control CAP256-VRC26.09 (VRC26.09). Bess1-8, ElsE1-11 mAbs, and CAP256-VRC26.09 showed no detectable binding to BG505 gp120. (TIF) [file ppat.1012042.s006.tif]

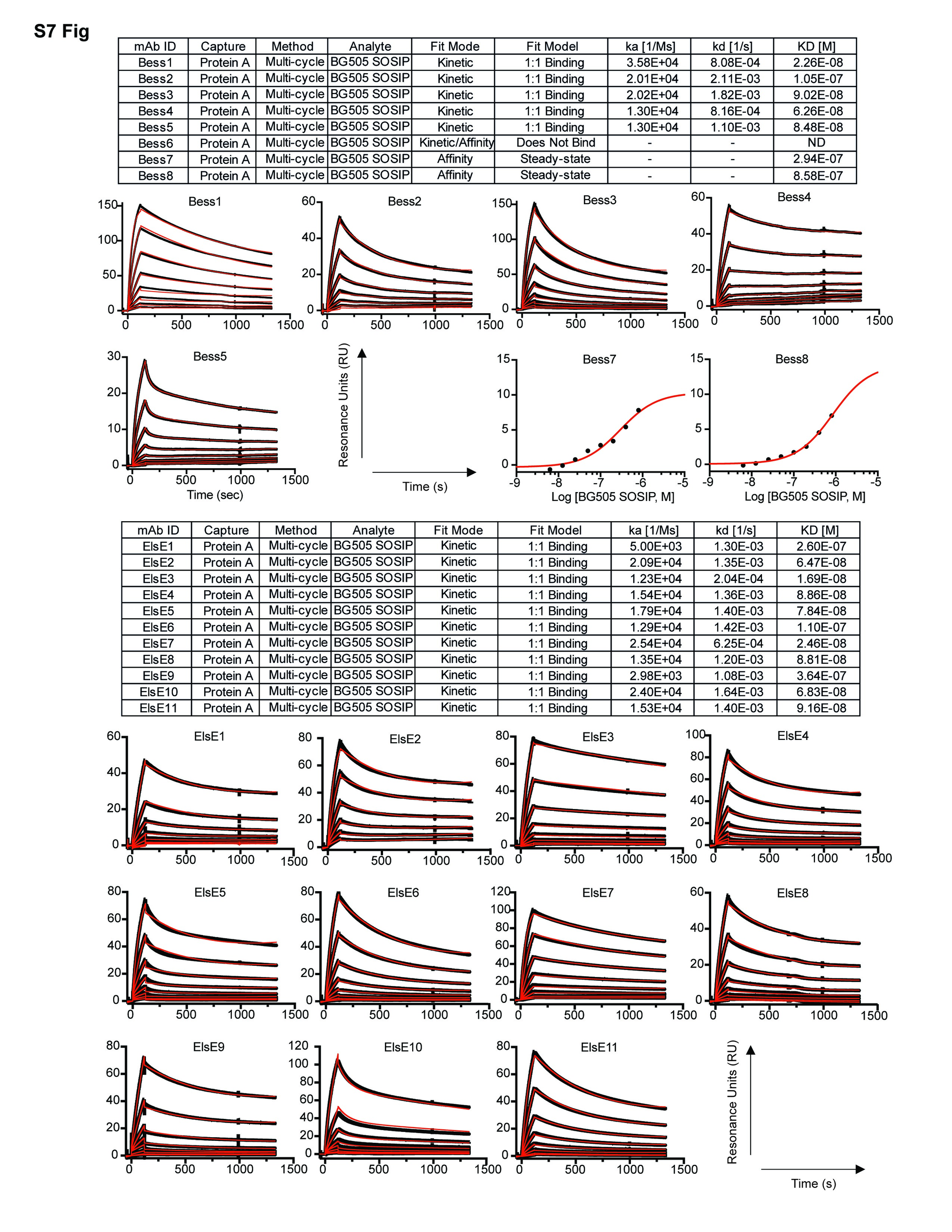

Supplement: S7 Fig — Summarized results of BG505 binding to Bess and ElsE mAbs via Protein A capture, multi-cycle method. SPR experiments are shown in black and best global fits are shown in red. 1:1 Langmuir binding model and stead-state were used to calculate the association (ka) and dissociation (kd) rate constants where indicated. Experimental traces obtained from SPR sensograms for BG505 SOSIP binding to mAbs are also shown. SPR experiments are represented in black and best global fits are indicated in red. 1:1 Langmuir binding model and steady-state were used to calculate the association (ka) and dissociation (kd) rate constants. (TIF) [file ppat.1012042.s007.tif]

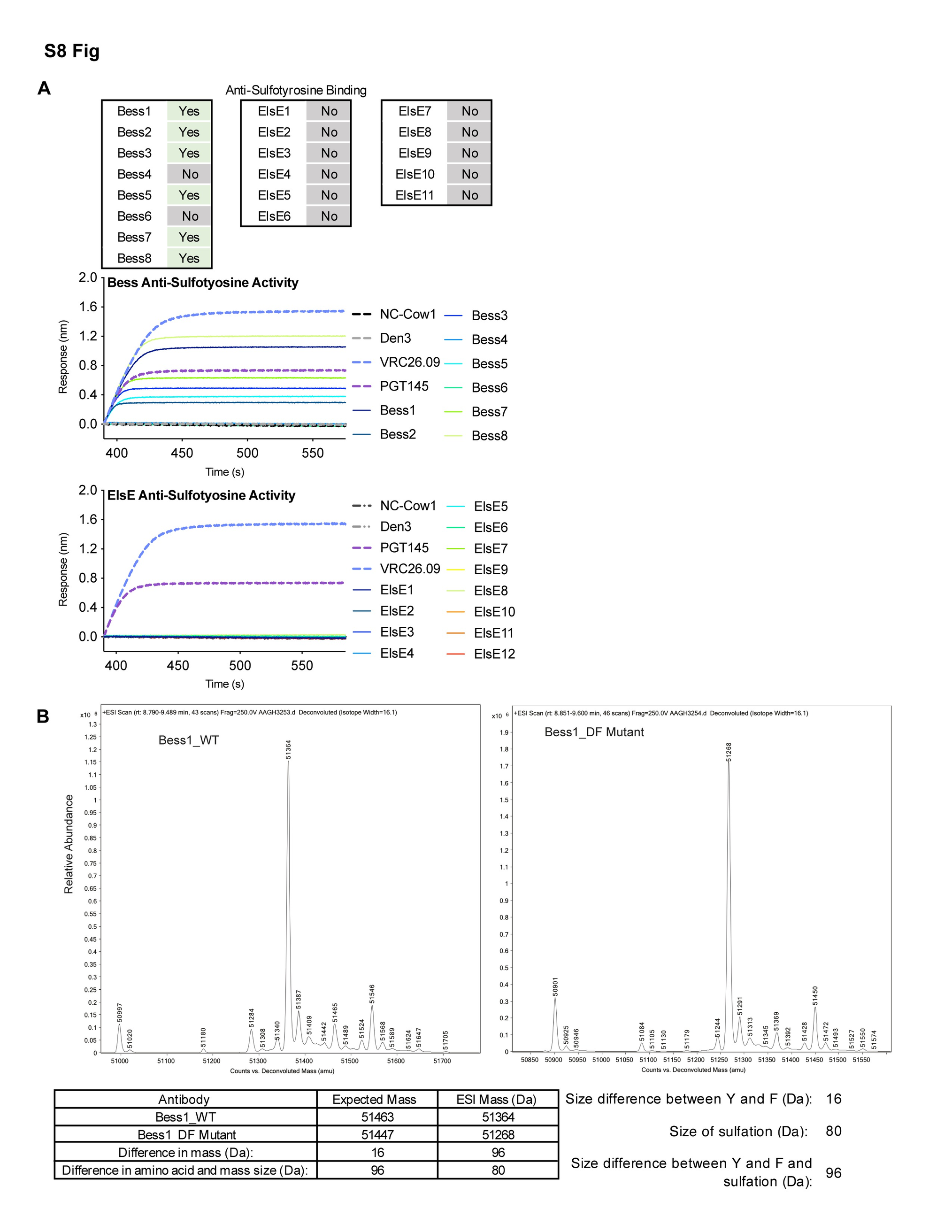

Supplement: S8 Fig — (A) Bess and ElsE series mAbs were evaluated for their ability to bind to a mouse derived anti-sulfotyrosine antibody using BLI. NC-Cow1 and Den3 were used as negative controls. CAP256-VRC26.09 and PGT145 were used as positive controls. The table showing which antibodies are bound is shown on the top. Positive and negative control lines are shown as dashes on the bottom. (B) To evaluate the loss of tyrosine sulfation, ESI mass spectrometry was used to compare the mass difference of Bess1_WT and Bess1_DF Mutant. ESI mass spectrometry spectra are shown. Below is a chart with a comparison of the mass differences and expectations for the mAbs, amino acids, and tyrosine sulfation sizes sulfation are summarized at the bottom. Expected mass is the calculated amino acid mass for the WT and DF mutant respectively, differences are due to presence or absence of tyrosine sulfation. (TIF) [file ppat.1012042.s008.tif]

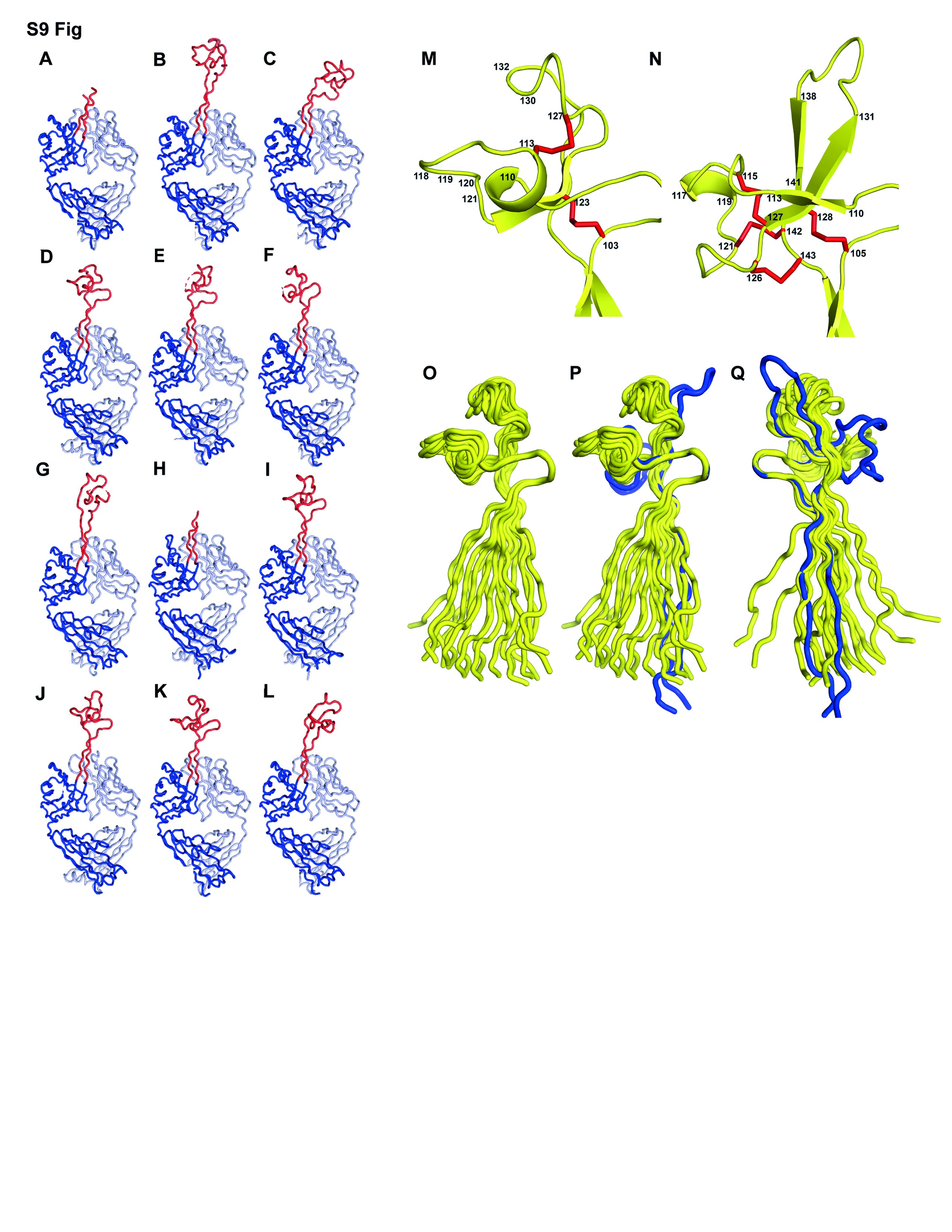

Supplement: S9 Fig — The light and heavy chains are colored light and dark blue, respectively, with the ultralong CDR H3s highlighted in red. Two structures (ElsE8 and ElsE11) contain two Fabs in the crystallographic asymmetric unit, with both shown here. CDR H3 knob regions have very weak electron density in the Bess4 and ElsE8 mol1 structures that are not modeled. Extreme flexibility in the stalk regions is evident among these structures, for example when comparing the two Fab molecules in the asymmetric unit for ElsE11, where the knob domains are rotated approximately 114° from each other. (A) Fab Bess4, 2.8Å, (B) Fab Bess7, 2.1Å, (C) ElsE1, 1.81Å, (D) ElsE2,1.90Å, (E) ElsE5, 1.89Å, (F) ElsE6, 2.35Å, (G) ElsE7, 2.54Å, (H) ElsE8 mol1, 1.83Å, (I) ElsE8 mol2, 1.83Å, (J) ElsE9, 2.3Å, (K) ElsE11 mol1, 2.65Å, (L) ElsE11 mol2, 2.65Å. (M) The knob of Fab ElsE1 is shown with secondary structural elements highlighted with cartoon strands or helices. There are 2 disulfide bonds in the ElsE family of Fabs, linking residues 103–123 and 113–127 (red). ElsE1 has a short α-helical region between residues 110–113, and a short 310 helical region between residues 130–132. In addition to the canonical type II turn are residues 105–106 (i+1, i+2) there is a type II turn at residues 118–119 (i+1, i+2) followed immediately by a type I turn around residues 120–121 (i+1, i+2). (N) The knob of Bess7 is very different from those of the ElsE family of Fabs, with 4 disulfide bonds between residues 105–128, 115–127, 121–142, and 126–143. There is a short helical turn between residues 117–119. (O) The ultralong CDR H3’s from the ElsE Fabs (ElsE1, ElsE2, ElsE5, ElsE6, ElsE7, ElsE8, ElsE9, ElsE11) with ordered knob electron density are superimposed by their CPDG type II beta turn motifs. These CDR knobs are all highly similar (see Fig 4C for sequences) in sequence and structure. (P) Same as for (O), however the Bess7 CDR H3 (blue) has been included in the superposition to show the difference in its knob region. (Q) [file ppat.1012042.s009.tif]

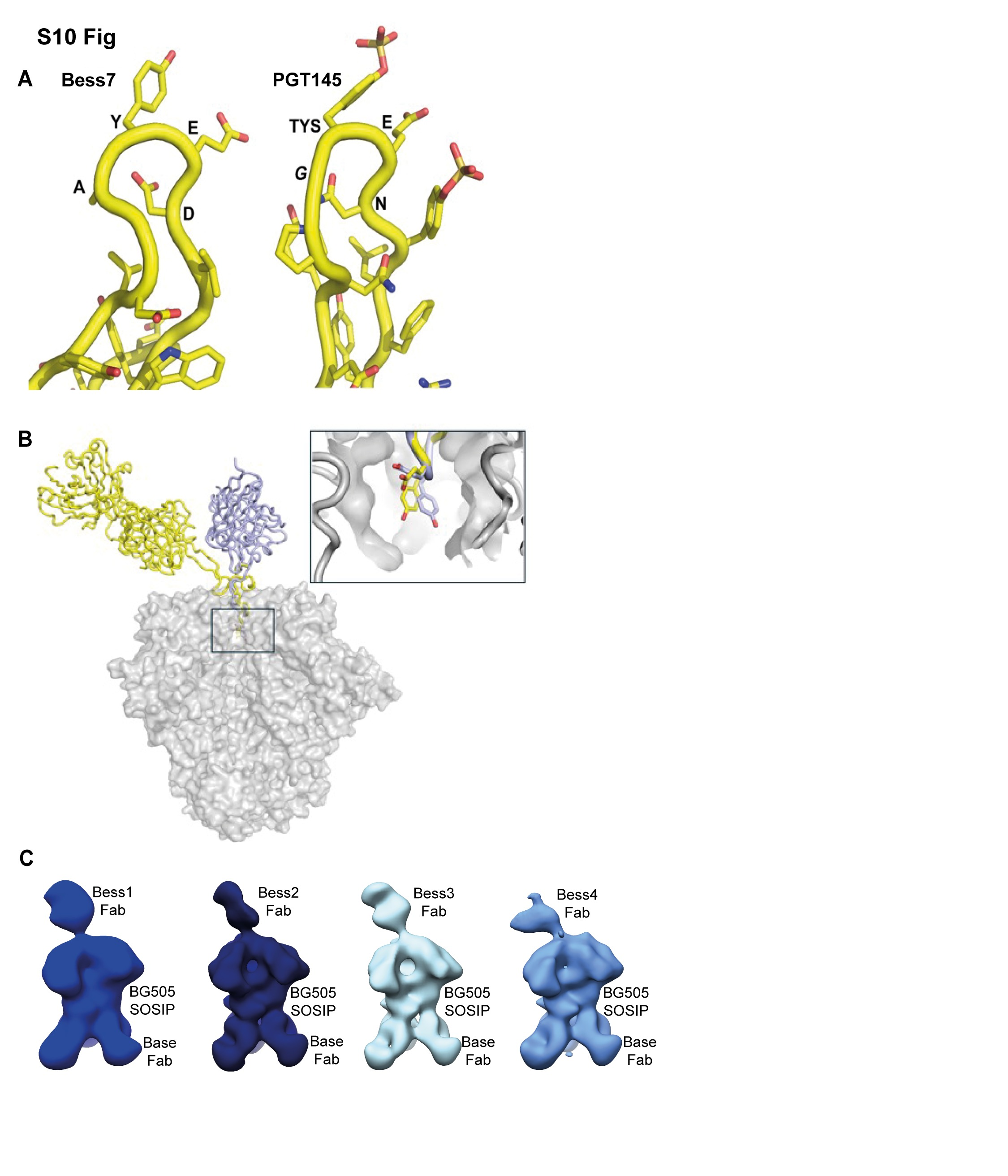

Supplement: S10 Fig — (A) Structural similarities of the Bess7 and PGT145 CDRH3 regions. The Loop region of the Bess7 knob folds into a 14 residues β-hairpin, with i-i+3 residues DEYA at the distal tip. The long CDRH3 of human anti-HIV Fab PGT145 has a similar type I β-turn at its tip, with i-i+3 residues NETysG. (B) The Bess7 Fab was superimposed onto Fab PGT145 bound to an HIV trimer (PDB 5V8L) using residues 134–135 (Tyr-Glu) from the Bess7 knob and residues 100H-100I (Tys-Glu) from PGT145. Bess7 is shown in yellow, PGT145 in light blue, and the trimer as a solid gray surface. An enlargement of the residues used for the superposition is shown in the upper right panel. The long Bess7 stalk might enable the body of the Bess7 Fab to avoid clashes with the trimer. While Tys100H in PGT145 is sulfated, we did not see convincing electron density for sulfation on the Bess 7 residue Tyr 134. Potential flexibility in the Bess7 stalk region has not been considered in this very simple model. (C) Negative stain 3D reconstructions of Bess1, Bess2, Bess3 or Bess4 in complex with BG505 SOSIP and base-binding Fab RM20A3 (added for angular sampling. (TIF) [file ppat.1012042.s010.tif]

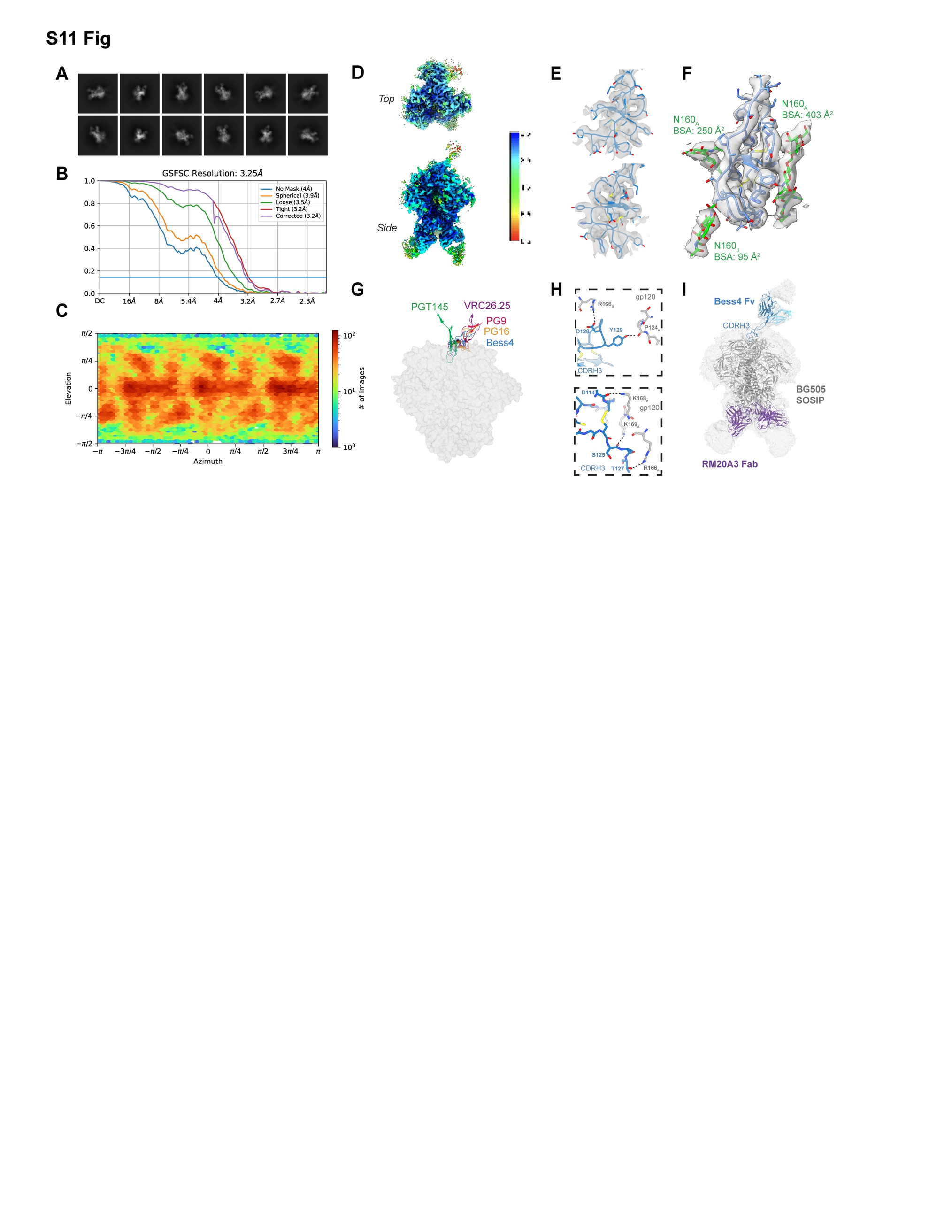

Supplement: S11 Fig — (A) Representative 2D class averages. (B) Fourier Shell Correlation resolution estimation. (C) Angular distribution. (D) Local resolution estimation (Å) of the Bess4-BG505-RM20A3 cryo-EM dataset. (E) EM map density (contoured at 5σ) and modeled residues of CDRH3. (F) EM map density (contoured at 5σ) with a focus on CDRH3 interactions with Env N160 glycans. Calculated buried surface area (BSA) between CDRH3 and modeled glycans from each protomer are listed. (G) Overlay of CDRH3 from Bess4 and four human bnAbs, with BG505 SOSIP trimer shown as surface transparency. (H) Predicted hydrogen bonds and salt bridges between Bess4 and BG505 SOSIP. (I) Unsharpened and low contour (2σ) map of Bess4-BG505-RM20A3 with the Fv portion of the Bess4 crystal structure docked in. (TIF) [file ppat.1012042.s011.tif]
