# Supplementary material for: Immunization of cows with HIV envelope trimers generates broadly neutralizing antibodies to the V2-apex from the ultralong CDRH3 repertoire
Source: PLoS Pathog. 2024 Sep 9;20(9):e1012042. doi: 10.1371/journal.ppat.1012042 (PMC11412654; doi:10.1371/journal.ppat.1012042)
Supplement: S1 Table — ID50 values are shown as 1/dilution. (PDF) [file ppat.1012042.s013.pdf]

S1 Table: Neutralization ID_{50} titers are shown for IgG purified from sera tested against murine leukemia virus (MLV) for all four cows over the course of immunization. ID_{50} values are shown as 1/dilution.

MLV				
ID_{50} (1/dilution)	Group 1		Group 2	
Day	Cow-485	Cow-16157	Cow-488	Cow-491
59	<35	<35	<35	<35
65	<35	<35	<35	<35
72	<35	<35	<35	<35
80	<35	37	<35	<35
85	<35	<35	<35	<35
150	<35	303	<35	<35
157	<35	70	<35	<35
164	<35	49	<35	<35
171	<35	50	<35	<35
234	<35	175	<35	<35
346	<35	<35	<35	<35
352	<35	<35	<35	<35
359	<35	<35	<35	<35

Neutralization ID_{50} (1/dilution)		
35	150	300

S2 Table: Neutralization ID_{50} titers are shown for IgG purified from sera from Day 359 of all four cows. Geomean ID_{50} values and percent breadth are shown at the bottom of the graph. ID_{50} values are shown as 1/dilution.

ID_{50} (1/dilution)		Group1		Group2	
Virus	Clade	Cow-485	Cow-16157	Cow-488	Cow-491
246F3	AC	<35	<35	297	<35
25710	C	1521	76	15193	<35
398F1	A	231	<35	234	118
BJOX2000	BC	<35	<35	718	<35
CE1176	C	68	72	672	<35
CE0217	C	45	<35	437	<35
CH119	BC	<35	<35	143	<35
CNE55	AE	4258	228	11923	70
CNE8	AE	581	131	4048	1094
Tro.11	B	<35	74	59	47
X1632	G	169	<35	461	46
X2278	B	<35	<35	65	<35
Geomean ID_{50}		333	104	612	114
% Breadth		58%	42%	100%	42%

Neutralization ID_{50} (1/dilution)				
35	150	500	1000	5000

S3 Table: Neutralization IC_{50} (μg/ml) and ID_{50} (1/dilution) titers are shown for ID_{50} (1/dilution) and IgG purified from sera from Day 359 for cow-485 and cow-488. Geomean IC_{50} and ID_{50} values are shown at the bottom of the graph.

Virus Strain	Clade	Bess1	Bess2	Bess4	ElsE1	ElsE2	Cow-485	Cow-488
6535.3	B	>50	>50	>50	>50	>50	<35	122
SC422661.8	B	>50	>50	>50	>50	>50	36	<35
PVO.4	B	>50	>50	>50	>50	>50	<35	<35
TRO.11	B	>50	>50	>50	>50	>50	<35	<35
AC10.0.29	B	>50	>50	>50	>50	>50	<35	<35
RHPA4259.7	B	>50	>50	>50	>50	>50	<35	<35
TRJO4551.58	B	>50	>50	>50	>50	>50	<35	<35
WITO4160.33	B	>50	>50	>50	>50	>50	<35	94
CAAN5342.A2	B	>50	>50	>50	>50	>50	<35	<35
WEAU_d15_410_5017	B (T/F)	>50	>50	>50	>50	>50	<35	<35
1006_11_C3_1601	B (T/F)	>50	>50	>50	>50	>50	<35	<35
1056_10_TA11_1826	B (T/F)	>50	>50	>50	>50	>50	<35	43
1012_11_TC21_3257	B (T/F)	>50	>50	>50	>50	>50	<35	56
6240_08_TA5_4622	B (T/F)	>50	>50	>50	>50	>50	<35	<35
6244_13_B5_4576	B (T/F)	>50	>50	>50	>50	>50	<35	<35
62357_14_D3_4589	B (T/F)	>50	>50	>50	>50	>50	<35	<35
Du156.12	C	0.016	0.084	0.006	0.004	0.001	120	11126
ZM197M.PB7	C	>50	20	>50	36	0.607	<35	114
ZM214M.PL15	C	>50	>50	>50	>50	>50	318	<35
ZM233M.PB6	C	>50	5	0.508	0.119	0.100	95	264
ZM249M.PL1	C	0.014	0.029	0.015	0.450	0.032	92	318
ZM53M.PB12	C	>50	>50	0.014	10	0.049	98	280
ZM109F.PB4	C	>50	0.496	0.056	0.084	0.020	224	1485
ZM135M.PL10a	C	>50	>50	>50	>50	>50	<35	<35
CAP45.2.00.G3	C	0.001	0.002	0.0006	0.027	0.0006	2126	5804
CAP210.2.00.E8	C	>50	13	0.026	0.289	0.078	<35	154
HIV-0013095-2.11	C	>50	>50	0.023	0.043	0.007	38	6901
HIV-16055-2.3	C	0.001	0.014	0.0006	0.004	0.0006	1017	14435
Ce0393_C3	C (T/F)	>50	0.302	0.008	0.387	0.033	241	25670
Ce1176_A3	C (T/F)	>50	>50	0.049	8	0.004	68	672
Ce2010_F5	C (T/F)	>50	>50	>50	>50	>50	<35	<35
Ce0682_E4	C (T/F)	>50	>50	>50	>50	>50	<35	40
Ce1172_H1	C (T/F)	>50	>50	0.045	0.968	0.033	50	358
Ce2060_G9	C (T/F)	0.190	50	>50	>50	2	412	218
Ce703010054_2A2	C (T/F)	>50	>50	>50	>50	>50	<35	<35
246F C1G	C (T/F)	0.001	0.003	>50	>50	>50	<35	58
249M B10	C (T/F)	0.028	0.118	0.044	1	0.083	69	345
ZM247v1(Rev-)	C (T/F)	11	6	5	>50	>50	<35	39
7030102001E5(Rev-)	C (T/F)	>50	>50	>50	>50	>50	<35	<35
1394C9G1(Rev-)	C (T/F)	0.551	0.168	0.249	2	0.004	2034	1345
Ce704809221_1B3	C (T/F)	>50	>50	>50	>50	>50	<35	<35
CNE19	BC	>50	>50	>50	>50	0.147	72	932
CNE20 (CRF07_BC)	BC	>50	>50	0.319	>50	0.299	<35	135
CNE17	BC	0.517	0.517	0.016	0.085	0.017	140	851

CNE30	BC	>50	>50	>50	>50	>50	<35	<35
CNE52	BC	0.290	0.290	>50	0.937	0.020	<35	498
CNE53	BC	>50	>50	>50	>50	>50	<35	<35
CNE58	BC	0.458	1	0.087	4	0.044	494	904
MS208.A1	A	>50	>50	18	0.566	0.082	<35	582
Q23.17	A	1	0.163	0.006	36	2	48	<35
Q461.e2	A	0.025	0.172	0.075	1	0.105	<35	321
Q769.d22	A	>50	>50	0.001	1	0.078	112	277
Q259.d2.17	A	0.009	0.016	0.127	7	0.024	<35	606
0330.v4.c3	A	11	0.167	0.105	0.145	0.0006	<35	4512
0260.v5.c36	A	>50	0.373	0.091	39	18	<35	<35
191955_A11	A (T/F)	0.005	0.007	0.374	1	0.001	1773	780
T257-31	CRF02_AG	0.035	0.057	0.003	0.027	0.001	2845	84
263-8	CRF02_AG	0.025	0.335	0.040	0.186	0.024	506	<35
T250-4	CRF02_AG	0.0006	0.002	0.067	0.005	0.006	6756	10458
T251-18	CRF02_AG	>50	>50	>50	>50	>50	<35	<35
T278-50	CRF02_AG	>50	>50	>50	14	1	<35	<35
T255-34	CRF02_AG	>50	>50	>50	>50	>50	<35	<35
211-9	CRF02_AG	>50	>50	>50	>50	>50	<35	<35
235-47	CRF02_AG	>50	>50	>50	24	3	<35	<35
620345.c01	CRF01_AE	0.080	0.055	0.001	0.087	0.006	160	2238
C1080.c03	CRF01_AE	>50	5	0.014	0.0006	0.0006	15010	145536
R2184.c04	CRF01_AE	0.062	0.654	0.007	0.481	0.024	168	547
R1166.c01	CRF01_AE	>50	>50	>50	>50	>50	393	151
R3265.c06	CRF01_AE	>50	1	0.014	0.096	0.016	671	611
C3347.c11	CRF01_AE	12	0.545	>50	>50	>50	<35	<35
C4118.c09	CRF01_AE	0.005	0.021	0.0006	0.0006	0.0006	1403	11276
CNE8	CRF01_AE	0.386	0.057	0.263	0.029	0.005	581	4048
CNE5	CRF01_AE	0.001	0.010	0.184	0.027	0.0006	2300	5281
BJOX009000.02.4	CRF01_AE	>50	>50	>50	>50	>50	<35	58
BJOX015000.11.5	CRF01_AE (T/F)	>50	>50	>50	>50	>50	293	63
BJOX010000.06.2	CRF01_AE (T/F)	>50	>50	>50	>50	>50	<35	<35
BJOX025000.01.1	CRF01_AE (T/F)	>50	>50	2	>50	>50	268	53
X1193_C1	G	>50	>50	>50	>50	>50	<35	<35
P0402_C2_11	G	>50	>50	16	>50	26	<35	103
X1254_C3	G	>50	>50	>50	>50	>50	<35	<35
X2088_C9	G	>50	>50	0.017	0.087	0.022	456	2122
X2131_C1_B5	G	>50	>50	>50	>50	>50	<35	<35
P1981_C5_3	G	0.119	3	0.031	0.112	0.007	78	1945
X1632_S2_B10	G	11	>50	>50	0.231	0.024	169	461
3016.v5.c45	D	>50	26	>50	37	0.384	<35	35
A07412M1.vrc12	D	0.582	0.993	50	3	0.237	41	261
231965.c01	D	>50	>50	0.040	>50	0.088	<35	327
231966.c02	D	>50	>50	>50	>50	>50	<35	108
3817.v2.c59	CD	0.034	2	0.018	0.401	0.0006	197	7236
6480.v4.c25	CD	>50	>50	>50	>50	>50	<35	<35
6952.v1.c20	CD	>50	>50	>50	>50	>50	<35	<35
6811.v7.c18	CD	0.035	0.059	>50	>50	>50	<35	<35
89-F1_2_25	CD	>50	>50	>50	>50	7	<35	<35

3301.v1.c24	AC	0.245	>50	0.269	8	0.454		<35	45
6041.v3.c23	AC	0.018	0.086	>50	>50	>50		<35	<35
6545.v4.c1	AC	0.014	0.065	0.003	0.005	0.001		35	4006
0815.v3.c3	ACD	>50	>50	>50	>50	>50		<35	<35
3103.v3.c10	ACD	>50	>50	>50	>50	>50		<35	<35
	Geomean IC_{50}	0.061	0.238	0.047	0.352	0.031	Geomean ID_{50}	276	557

Neutralization IC_{50} (μg/ml)				
50	1	0.5	0.05	0.005

Neutralization ID_{50} (1/Dilution)				
35	150	500	1000	5000

S4 Table: Table of recovered heavy chains tested with native and universal light chains.

Sort Stats and Summary of Screening			Sort 1	Sort 2	Sort 3	Sort 4	Short CDRH3 (0-24 AA)	Long CDRH3 (25-49 AA)	Ultralong CDRH3 (50+ AA)
Cow 485	All	Cells Sorted	108	453	559	438	NA	NA	NA
		PCR Wells Positive for Heavy Chains	34	75	169	52	NA	NA	NA
		Heavy Chain Sequences Recovered	13	43	47	49	42	72	38
	Universal Light Chain	Tested IGHV-1*7 Heavy Chains	10	40	30	NT	0	52	28
		Expressed in Screen	9	22	25	NT	0	32	24
		Positive for BG505 Binding	8	13	12	NT	0	14	19
		Monoclonals with Cross-Clade Neutralization	1	1	4	2	0	0	8
	Native Light Chain	Heavy Chain/Light Chain Pairs	12	32	41	49	41	63	30
		Expressed in Screen	9	24	34	45	34	53	25
		Positive for BG505 Binding	7	12	12	20	13	22	16
		Monoclonals with Cross-Clade Neutralization	1	0	3	2	0	0	6
Cow 488	All	Cells Sorted	33	342	636	283	NA	NA	NA
		PCR Wells Positive for Heavy Chains	6	13	58	54	NA	NA	NA
		Heavy Chain Sequences Recovered	1	6	17	49	34	4	35
	Universal Light Chain	IGHV-1*7 Heavy Chains	0	2	1	NT	1	2	0
		Expressed in Screen	0	2	0	NT	0	2	0
		Positive for BG505 Binding	0	2	0	NT	0	2	0
		Monoclonals with Cross-Clade Neutralization	0	0	0	11	0	0	0
	Native Light Chains	Heavy Chain/Light Chain Pairs	1	5	16	49	33	3	35
		Expressed in Screen	1	5	10	44	25	3	32
		Positive for BG505 Binding	0	3	3	33	8	3	28

S5 Table: ElsE and Bess antibodies were tested for their ability to neutralize the 12-virus global panel. IC_{50} (μg/ml), IC_{80} (μg/ml), and MPN (%) are shown for all antibodies whose IC_{50} reach at least 50% neutralization. MPN= Maximum Percent Neutralized.

		398F1	TRO.11	X2278	25710	CE0217	Ce1176	X1632	246F3	CNE55	CNE8	BJOX	CH119
		A	B	B	C	C	C	G	AC	AE	AE	BC	BC
Bess1	IC_{50} (μg/ml)	>50	>50	>50	<.005	>50	>50	11	0.054	<.005	0.386	0.066	>50
	IC_{80} (μg/ml)	>50	>50	>50	0.013	>50	>50	>50	0.215	<.005	2	0.315	>50
	MPN	<50	<50	<50	100%	<50	<50	58%	94%	100%	90%	94%	<50
Bess2	IC_{50} (μg/ml)	>50	>50	>50	0.014	0.355	>50	>50	0.03	<.005	0.057	0.232	>50
	IC_{80} (μg/ml)	>50	>50	>50	0.057	1	>50	>50	0.121	0.019	0.229	0.928	>50
	MPN	<50	<50	<50	99%	88%	<50	<50	99%	100%	99%	98%	<50
Bess3	IC_{50} (μg/ml)	>50	>50	>50	0.067	>50	>50	6	0.063	0.042	0.603	0.067	>50
	IC_{80} (μg/ml)	>50	>50	>50	0.268	>50	>50	>50	0.250	0.170	2	0.270	>50
	MPN	<50	<50	<50	100%	<50	<50	62%	99%	100%	97%	99%	<50
Bess4	IC_{50} (μg/ml)	>50	>50	>50	0.064	0.127	0.049	>50	0.245	0.015	0.263	0.025	>50
	IC_{80} (μg/ml)	>50	>50	>50	0.258	0.509	0.195	>50	0.982	0.061	1	0.098	>50
	MPN	<50	<50	<50	98%	95%	93%	<50	92%	100%	100%	99%	<50
Bess5	IC_{50} (μg/ml)	>50	>50	>50	>50	24	13	>50	>50	0.02	0.093	14	>50
	IC_{80} (μg/ml)	>50	>50	>50	>50	>50	>50	>50	>50	0.079	1	>50	>50
	MPN	<50	<50	<50	<50	50%	56%	<50	<50	97%	91%	62%	<50
Bess6	IC_{50} (μg/ml)	>50	>50	49	41	>50	17	>50	>50	>50	>50	>50	>50
	IC_{80} (μg/ml)	>50	>50	>50	>50	>50	34	>50	>50	>50	>50	>50	>50
	MPN	<50	<50	52%	58%	<50	87%	<50	<50	<50	<50	<50	<50
Bess7	IC_{50} (μg/ml)	>50	>50	>50	0.477	>50	>50	>50	>50	<.005	>50	31	>50
	IC_{80} (μg/ml)	>50	>50	>50	<50	>50	>50	>50	>50	0.02	>50	<50	>50
	MPN	<50	<50	<50	80%	<50	<50	<50	<50	98%	<50	50%	<50
Bess8	IC_{50} (μg/ml)	>50	>50	>50	0.009	>50	>50	>50	2	0.007	9	0.21	>50
	IC_{80} (μg/ml)	>50	>50	>50	0.525	>50	>50	>50	7	0.029	>50	0.839	>50
	MPN	<50	<50	<50	97%	<50	<50	<50	72%	97%	59%	84%	<50
ElsE1	IC_{50} (μg/ml)	>50	>50	>50	<.005	2	8	0.231	0.058	<.005	0.029	0.033	>50
	IC_{80} (μg/ml)	>50	>50	>50	0.019	>50	>50	0.924	0.233	0.019	0.116	0.133	>50
	MPN	<50	<50	<50	99%	74%	66%	81%	88%	99%	100%	100%	<50
ElsE2	IC_{50} (μg/ml)	>50	>50	>50	0.006	0.009	<.005	0.024	0.007	<.005	0.005	0.023	>50
	IC_{80} (μg/ml)	>50	>50	>50	0.023	0.037	0.018	0.096	0.027	0.014	0.022	0.091	>50
	MPN	<50	<50	<50	100%	94%	94%	84%	96%	99%	100%	100%	<50
ElsE3	IC_{50} (μg/ml)	>50	>50	>50	<.005	0.009	0.062	0.161	0.153	0.007	0.009	0.026	>50
	IC_{80} (μg/ml)	>50	>50	>50	0.015	0.035	0.249	0.643	0.611	0.027	0.034	0.103	>50
	MPN	<50	<50	<50	100%	97%	92%	84%	90%	98%	100%	100%	<50
ElsE4	IC_{50} (μg/ml)	>50	>50	>50	0.016	23	>50	1	2	<.005	0.011	0.035	>50
	IC_{80} (μg/ml)	>50	>50	>50	0.062	>50	>50	>50	>50	0.012	0.042	0.139	>50
	MPN	<50	<50	<50	98%	54%	<50	74%	78%	98%	100%	100%	<50
ElsE5	IC_{50} (μg/ml)	>50	>50	>50	0.008	26	>50	3	3	0.008	0.011	0.031	>50
	IC_{80} (μg/ml)	>50	>50	>50	0.032	>50	>50	>50	>50	0.031	0.044	0.123	>50
	MPN	<50	<50	<50	99%	52%	<50	68%	74%	98%	100%	99%	<50
ElsE6	IC_{50} (μg/ml)	>50	>50	>50	0.06	>50	>50	5	>50	0.033	0.023	0.013	>50
	IC_{80} (μg/ml)	>50	>50	>50	0.238	>50	>50	>50	>50	0.13	0.093	0.051	>50
	MPN	<50	<50	<50	91%	<50	<50	68%	<50	95%	99%	99	<50
ElsE7	IC_{50} (μg/ml)	>50	>50	>50	0.007	0.77	5	0.022	2	<.005	0.007	0.019	>50
	IC_{80} (μg/ml)	>50	>50	>50	0.028	3	>50	0.087	>50	0.019	0.026	0.076	>50
	MPN	<50	<50	<50	99%	80%	68%	91%	78%	99%	99%	100%	<50
ElsE8	IC_{50} (μg/ml)	>50	>50	>50	0.023	3	10	0.654	0.056	0.024	0.023	0.067	>50
	IC_{80} (μg/ml)	>50	>50	>50	0.094	>50	>50	>50	0.223	0.096	0.092	0.267	>50
	MPN	<50	<50	<50	99%	71%	60%	74%	90%	97%	99%	100%	<50
ElsE9	IC_{50} (μg/ml)	>50	>50	>50	0.017	0.723	3	0.067	0.047	0.006	0.019	0.043	>50
	IC_{80} (μg/ml)	>50	>50	>50	0.067	>50	>50	0.269	0.188	0.022	0.077	0.172	>50
	MPN	<50	<50	<50	99%	75%	69%	81%	92%	99%	100%	100%	<50
ElsE10	IC_{50} (μg/ml)	>50	>50	>50	0.025	>50	17	11	>50	0.009	0.063	0.093	>50
	IC_{80} (μg/ml)	>50	>50	>50	0.102	>50	>50	>50	>50	0.12	0.167	0.372	>50
	MPN	<50	<50	<50	93%	<50	60%	62%	<50	96%	98%	93%	<50
ElsE11	IC_{50} (μg/ml)	>50	>50	>50	0.033	>50	>50	0.772	>50	0.016	0.031	0.027	>50
	IC_{80} (μg/ml)	>50	>50	>50	0.134	>50	>50	>50	>50	0.064	0.161	0.283	>50
	MPN	<50	<50	<50	93%	<50	<50	72%	<50	96%	98%	99%	<50

S6 Table: X-ray data collection and refinement statistics for Bess and ElsE Fabs.

	Fab ElsE1	Fab ElsE2	Fab ElsE5	Fab ElsE6	Fab ElsE7	Fab ElsE8	Fab ElsE9	Fab ElsE11	Fab Bess4	Fab Bess7
**PDB code**	8V4I	8VBJ	8VBK	8VBL	8VBM	8VBN	8VBO	8VBR	8VBP	8VBQ
**Crystallization condition**	40% ethylene glycol, 0.1M HEPES, pH 7.5, 5% Peg3000, 20°	0.1M Tris, pH 8.5, 1.5M ammonium sulfate, 12% glycerol, 4°	0.1M sodium cacodylate, pH 6.5, 40% MPD, 5% Peg8000, 20°	0.1M Ches, pH 9.5, 50% Peg200, 20°	0.1M Ches, pH 9.5, 20% Peg 8000, 4°	20% Peg3350, 0.2M KCl, 4°	0.1M sodium cacodylate, pH 6.5, 40% MPD, 5% Peg 8000, 20°	0.1M Ches, pH 9.5, 50% Peg200, 4°	70% MPD, 0.1M Hepes, pH 7.5, 20°	0.095M sodium citrate, pH 5.6, 19% 2-propanol, 5% glycerol, 19% Peg4000, 20°
**Wavelength**	0.97946	0.97946	1.03317	0.97946	0.97946	0.97741	0.97946	0.97741	0.97946	0.97741
**Beamline**	SSRL 12-1	SSRL 12-2	APS 23-ID-B	SSRL 12-2	SSRL 12-2	ALS 5.0.1	SSRL 12-1	ALS 5.0.1	SSRL 12-1	ALS 5.0.1
**Resolution range**	37.89 - 1.81 (1.88 - 1.81)	45.48 - 1.90 (1.97 - 1.90)	43.86 - 1.89 (1.96 - 1.89)	46.80 - 2.35 (2.43 - 2.35)	42.23 - 2.54 (2.63 - 2.54)	46.95 - 1.83 (1.90 - 1.83)	36.30 - 2.30 (2.38 - 2.30)	49.58 - 2.65 (2.75 - 2.65)	29.94 - 2.80 (2.90 - 2.80)	48.29 - 2.10 (2.18 - 2.10)
**Space group/twin law**	$C\ 2\ 2\ 2_1$	$C\ 2\ 2\ 2_1$	$P\ 2_12_12_1$	$P\ 2_1\ 2_1\ 2$	$P\ 2_1\ 2_1\ 2_1$	$P\ 2_1\ 2_1\ 2_1$	$P\ 2_1\ 2_1\ 2$	$P\ 2_1 2_1\ 2_1$ l,h,-k	$P\ 2_1\ 2_1\ 2_1$	C 2
**Unit cell (Å,°)**	69.79, 84.12, 174.58, 90, 90, 90	69.96, 178.59, 105.71, 90, 90, 90	56.26, 70.02, 109.08, 90, 90, 90	117.59, 69.98, 77.32, 90, 90, 90	58.22, 70.61, 123.97, 90, 90, 90	59.22, 120.27, 150.30, 90, 90, 90	70.11, 150.89, 51.32, 90, 90, 90	70.05, 70.19, 241.83, 90, 90, 90	66.61, 70.28, 111.46, 90, 90, 90	83.00, 69.78, 87.52, 90, 103.52, 90
**Total reflections**	460,645 (32,773)	442,490 (44,977)	423,944 (39,845)	168,777 (17,085)	76,645 (6407)	1,219,769 (90,327)	81,165 (7982)	378,268 (10,647)	44,117 (4130)	98,588 (7823)
**Unique reflections**	45,028 (3292)	51,939 (5160)	34,653 (3286)	27,185 (2649)	16,971 (1511)	95,768 (9306)	24,379 (2325)	33,457 (1618)	12,710 (1143)	28,113 (2607)
**Multiplicity**	10.2 (10.0)	8.5 (8.7)	12.2 (12.1)	6.2 (6.4)	4.5 (4.2)	12.8 (9.8)	3.3 (3.4)	11.3 (6.6)	3.5 (3.3)	3.5 (3.0)
**Completeness (%)**	95.6 (70.1)	98.8 (99.2)	98.7 (94.9)	99.7 (98.8)	96.8 (84.6)	99.8 (98.2)	97.8 (95.3)	94.0 (63.9)	93.8 (87.2)	98.8 (92.4)
**Mean I/sigma(I)**	10.5 (0.9)	8.4 (1.4)	13.8 (2.4)	9.1 (2.4)	7.5 (1.5)	15.9 (1.3)	9.3 (2.5)	11.3 (6.6)	7.0 (3.0)	8.7 (1.2)
**Wilson B-factor**	34	23	27	34	44	26	44	51	38	32
**R-merge (%)**	15.5 (251)	22.4 (224)	11.8 (109)	19.8 (140)	16.4 (135)	11.6 (173)	9.2 (77.8)	9.0 (84.8)	19.1 (80.5)	15.2 (107)
**R-meas (%)**	16.2 (264)	23.8 (238)	12.3 (114)	21.7 (152)	18.6 (155)	12.0 (183)	10.8 (90.9)	9.4 (91.4)	22.4 (95.9)	17.9 (128)
**R-pim (%)**	4.6 (77.2)	7.9 (77.6)	3.5 (32.3)	8.6 (59.4)	8.6 (74.2)	3.3 (57.5)	5.6 (46.2)	2.7 (32.0)	11.5 (51.0)	9.3 (70.3)
**CC1/2 (%)**	99.6 (41.0)	99.5 (41.3)	99.8 (86.7)	99.3 (49.7)	99.0 (40.0)	99.9 (50.8)	99.5 (61.1)	99.0 (77.4)	97.3 (49.8)	99.2 (37.6)
**Reflections used in refinement**	44,983 (3264)	51,917 (5148)	34,637 (3277)	27,183 (2649)	16,886 (1437)	95,655 (9237)	24,379 (2325)	33,316 (2271)	12,577 (1141)	28,099 (2601)
**Reflections used for R-free**	2273 (190)	2580 (266)	1773 (159)	1331 (141)	783 (77)	1970 (191)	1175 (117)	1993 (138)	620 (62)	1348 (114)
**R-work (%)**	22.1 (41.1)	19.5 (35.0)	20.4 (33.9)	19.7 (27.7)	23.5 (37.8)	22.4 (37.5)	24.8 (34.1)	24.7 (49.7)	19.6 (26.2)	23.0 (32.5)
**R-free (%)**	25.7 (55.6)	22.9 (37.2)	24.5 (39.3)	24.4 (34.5)	29.0 (46.8)	25.8 (39.6)	28.0 (38.9)	26.0 (56.4)	23.2 (30.9)	26.7 (30.7)
**Number of non-hydrogen atoms**	3750	3889	3707	3593	3463	7146	3535	7026	3257	3715
**macromolecules**	3563	3555	3496	3477	3463	6755	3465	7026	3257	3569
**solvent**	191	334	211	116	0	681	70	0	0	142
**Protein residues**	479	477	473	475	472	915	469	956	441	486
**RMS(bonds)**	0.003	0.013	0.003	0.002	0.002	0.008	0.002	0.003	0.003	0.003

**RMS(angles)**	0.66	1.05	0.64	0.56	0.51	0.98	0.53	0.56	0.58	0.56
**Ramachandran favored, allowed, outliers (%)**	94.3, 5.3, 0.4	97.5, 2.5, 0.0	96.8, 3.2, 0.0	96.2,3.4, 0.4	94.6, 4.7, 0.6	96.56, 3.33, 0.11	94.58, 4.34, 1.08	95.25, 4.11, 0.63	95.17, 4.37, 0.46	95.23, 4.77, 0.00
**Rotamer outliers (%)**	1.94	2.18	0.99	1.23	1.75	1.79	1.76	1.84	1.58	0.73
**Clashscore**	3.3	1.9	1.3	3.2	4.6	2.4	3.7	2.9	2.2	1.7
**Average B-factor**	45	31	35	43	65	35	63	59	36	47
**macromolecules**	45	31	35	43	65	35	63	59	36	48
**solvent**	44	35	36	41	NA	37	72	NA	NA	37

Statistics for the highest-resolution shell are shown in parentheses.

S7 Table: Secondary structural elements in stalk/knob regions. #=residue number, AA=residue type, and SS=secondary structure type. DSSP was used to assign the secondary structure type for residues in stalk/knob regions from the crystal structures [86]. SS are designated as: α-helix: H, 3_{10} helix :G, extended strand: E, bend: S, hydrogen bonded turn: T. Blank fields have 'coil' secondary structure. Blank AA fields indicate unmodeled residues. Residues at the tips of the two knob 'loops' (114-118 and 127 -133 in the ElsE Fabs and 114-120 and 132-137 in Bess7) are highlighted in bold; these might be expected to interact with Env.

	Bess7		ElsE1		ElsE2		ElsE5		ElsE6		ElsE7		ElsE8		ElsE9		ElsE11_1		ElsE11_2	
#	AA	SS	AA	SS	AA	SS	AA	SS	AA	SS	AA	SS	AA	SS	AA	SS	AA	SS	AA	SS
92	C		C		C		C		C		C		C		C		C		C	
93	T		T		T		T		T		T		T		T		T		T	
94	T	E	T	E	T	E	T	E	T	E	T	E	A	E	T		T	E	T	
95	V	E	V	E	V	E	V	E	V	E	V	E	V	E	V	E	V	E	V	E
96	H	E	H	E	H	E	H	E	H	E	H	E	V	E	R	E	R	E	R	E
97	Q	E	Q	E	Q	E	Q	E	Q	E	Q	E	Q	E	Q	E	Q	E	Q	E
98	T	E	Q	E	Q	E	Q	E	Q	E	Q	E	E	E	Q	E	Q	E	Q	E
99	T	E	T	E	T	E	T	E	T	E	T	E	T	E	V	E	T	E	T	E
100	K	E	R	E	R	E	R	E	R	E	R	E	R	E	H	E	R	E	R	E
101	T		K	E	K	E	K	E	K	E	K	E	K	E	K	E	K	E	K	E
102	T		G	E	S	E	G	E	S	E	S	E	S	E	T	E	S	E	S	E
103	K	E	C		C		C		C		C		C		C		C		C	
104	E	E	P		P		P		P		P		P		P		P		P	
105	C		D	T	G	T	D	S	A	S	D	S	D	T	Q	S	D	T	D	T
106	P		G	T	G	T	G	S	G	S	G	S	G	T	G	S	G	T	G	T
107	E	T	W		Y		W		Y		W		W		W		W		W	
108	G	T	R	E	T	E	S	E	T	E	M	E	R	E	R	E	T	E	T	E
109	Y		F	E	F	E	F	E	L	E	F	E	F	E	F	E	L	E	L	E
110	N	E	G	H	G	G	G	T	A	T	G	S	G	T	G	H	A	H	A	H
111	W	E	W	H	Y	G	W	T	K	T	F	T	W	T	W	H	K	H	K	H
112	D		D	H	D	G	D	T	D	T	D	T	D	T	D	H	D	H	D	H
113	D	S	C	H	C	G	C	T	C	T	C		C	T	C	H	C	H	C	H
114	**G**		**G**	**T**	**G**	**T**	**G**	**T**	**G**				**G**	**T**	**G**	**T**	**G**	**T**	**G**	**T**
115	**C**		**F**		**F**		**F**						**F**		**F**		**F**		**F**	
116	**G**		**H**		**H**								**H**		**H**		**Y**		**Y**	
117	**S**	**G**	**G**	**S**	**G**	**S**							**G**	**S**	**G**	**S**	**G**	**S**	**G**	**S**
118	**E**	**G**	**Y**	**T**	**W**	**T**							**Y**	**T**	**Y**	**T**	**Y**	**T**	**Y**	**T**
119	**L**	**G**	G	T	G	T			G		G		G	T	G	T	G	T	G	T
120	**G**		Q	T	S	T	!		S	T	S		T	T	S	T	S	T	S	T
121	C		E	T	D	T	E		E	T	E	S	E	T	D	T	E	T	E	T
122	G	S	D	S	D	S	D		D	S	D	S	D	S	D	S	D		D	S
123	G	S	C	E	C	E	C	E	C	E	C	E	C	E	C	E	C	E	C	E
124	A	S	Y	E	Y	E	Y	E	Y	E	V	E	Y	E	Y	E	Y	E	Y	E
125	D	S	E	S	P	S	D	S	D	S	D	S	E	S	E	S	D	S	D	S
126	C	S	D		D	S	D		D		D		D		D	S	D		D	

**127**	C		**C**	**S**	**C**	**S**	**C**	**S**	**C**	**T**	**C**	**T**	**C**	**S**	**C**		**C**	**T**	**C**	**T**
**128**	C	E	**I**	**S**	**S**	**S**	**T**	**S**	**S**	**T**	**S**	**T**	**V**	**S**	**I**	**S**	**T**	**T**	**T**	**T**
**129**	W	E	**D**	**S**	**D**		**D**		**D**	**T**	**D**	**T**	**D**		**D**	**T**	**D**		**D**	
**130**	G	E	**I**	**G**	**I**	**T**	**I**	**T**	**I**	**S**	**I**	**S**	**I**	**T**	**I**	**T**	**I**	**T**	**I**	**S**
**131**	G	E	**L**	**G**	**L**	**T**	**L**	**T**	**L**	**S**	**L**	**S**	**L**	**T**	**L**	**S**	**L**	**T**	**L**	**T**
**132**	**V**	**E**	**S**	**G**	**N**		**S**	**S**	**S**	**S**	**S**	**S**	**S**	**T**	**S**	**S**	**S**	**T**	**S**	**T**
**133**	**D**	**E**	**S**	**S**	**S**	**T**	**S**		**S**		**A**	**S**	**S**		**S**	**S**	**S**	**S**	**S**	
**134**	**E**	**T**	Q		D	T	Q		H		Q		E		Q		N		N	
**135**	**Y**	**T**	T	E	V		T		T	E	T	E	T	E	T	E	T	E	T	E
**136**	**A**	**T**	L	E	V	B	L	B	L	E	L	E	V	E	L	E	L	E	L	E
**137**	**G**	**E**	S		G		S		S		S		S		S		S		S	
**138**	D	E	P		P		P		P		P		S		A		P		P	
**139**	L	E	T	E	I	E	T	E	T	E	I	E	T	E	E	E	T	E	T	E
**140**	Y	E	D	E	D	E	D	E	T	E	Y	E	D	E	D	E	T	E	T	E
**141**	S	E	A	E	T	E	T	E	T	E	T	E	R	E	T	E	T	E	T	E
**142**	C		Y	E	H	E	Y	E	Y	E	N	E	Y	E	Y	E	H	E	H	E
**143**	C		E	E	E	E	E	E	E	E	A	E	E	E	E	E	E	E	E	E
**144**	S	E	F	E	F	E	F	E	L	E	Y	E	L	E	L	E	F	E	F	E
**145**	V	E	H	E	H	E	H	E	H	E	H	E	H	E	H	E	N	E	N	E
**146**	A		V	E	V	E	V	E	V	E	V	E	V	E	V	E	V	E	V	E
**147**	H		D	E	D	E	D	E	D	E	D	E	D	E	D		D	E	D	
**148**	T	E	A	E	A	E	A	E	A	E	A	E	A	E	A		A	E	A	
**149**	Y	E	W		W		W		W		W		W		W		W		W	
**150**	E	E																		
**151**	W	E																		
**152**	Y	E																		
**153**	V	E																		
**154**	D	E																		
**155**	A	E																		
**156**	W																			

S8 Table: Cryo-EM data collection, refinement and validation statistics.

	BG505 SOSIP + Bess4 Fab + RM20A3 Fab (EMD-41498) (PDB: 8TQ1)
**Data collection and processing**	
Microscope	TFS Krios
Magnification	130,000x
Voltage (kV)	300
Electron exposure (e–/Å^2)	55.5
Defocus range (μm)	-0.7 to -1.7
Detector	Gatan K2 Summit
Recording mode	Counting
Pixel size (Å)	1.045
Symmetry imposed	C1
Micrographs (no.)	3,871
Initial particle images (no.)	494,075
Final particle images (no.)	82,374
Map resolution (Å)<br>FSC threshold	3.3<br>0.143
Map sharpening *B* factor (Å^2)	-83.5
Map pixel size (Å)	1.045
Map resolution range (Å)	2.4-5.0
**Refinement**	
Initial model used (PDB code)	6x9r
Model resolution (Å)<br>FSC threshold	3.3<br>0.5
Model resolution range (Å)	2.4-4.0
EMRinger score	3.95
Model composition<br>Non-hydrogen atoms<br>Protein residues<br>Ligands	 19,788<br>2,400<br>79
Mean *B* factors (Å^2)<br>Protein<br>Ligand	 71<br>96
R.m.s. deviations<br>Bond lengths (Å)<br>Bond angles (°)	 0.006<br>1.125
Validation<br>MolProbity score<br>Clashscore<br>Poor rotamers (%)	 1.11<br>1.49<br>0.19
Ramachandran plot<br>Favored (%)<br>Allowed (%)<br>Disallowed (%)	 96.54<br>3.46<br>0.00
