# Supplementary material for: Immunization of cows with HIV envelope trimers generates broadly neutralizing antibodies to the V2-apex from the ultralong CDRH3 repertoire
Source: PLoS Pathog. 2024 Sep 9;20(9):e1012042. doi: 10.1371/journal.ppat.1012042 (PMC11412654; doi:10.1371/journal.ppat.1012042)
Supplement: S2 Table. Neutralization ID50 titers are shown for IgG purified from sera from Day 359 of all four cows. Geomean ID50 values and percent breadth are shown at the bottom of the graph. ID50 values are shown as 1/dilution — (PDF) [file ppat.1012042.s014.pdf]

S2 Table: Neutralization ID$_{50}$ titers are shown for IgG purified from sera from Day 359 of all four cows. Geomean ID$_{50}$ values and percent breadth are shown at the bottom of the graph. ID$_{50}$ values are shown as 1/dilution.

ID$_{50}$ (1/dilution)		Group1		Group2	
Virus	Clade	Cow-485	Cow-16157	Cow-488	Cow-491
246F3	AC	<35	<35	297	<35
25710	C	1521	76	15193	<35
398F1	A	231	<35	234	118
BJOX2000	BC	<35	<35	718	<35
CE1176	C	68	72	672	<35
CE0217	C	45	<35	437	<35
CH119	BC	<35	<35	143	<35
CNE55	AE	4258	228	11923	70
CNE8	AE	581	131	4048	1094
Tro.11	B	<35	74	59	47
X1632	G	169	<35	461	46
X2278	B	<35	<35	65	<35
Geomean ID$_{50}$		333	104	612	114
% Breadth		58%	42%	100%	42%

Neutralization ID$_{50}$ (1/dilution)				
35	150	500	1000	5000
