# Supplementary material for: Immunization of cows with HIV envelope trimers generates broadly neutralizing antibodies to the V2-apex from the ultralong CDRH3 repertoire
Source: PLoS Pathog. 2024 Sep 9;20(9):e1012042. doi: 10.1371/journal.ppat.1012042 (PMC11412654; doi:10.1371/journal.ppat.1012042)
Supplement: S3 Table — Geomean IC50 and ID50 values are shown at the bottom of the graph. (PDF) [file ppat.1012042.s015.pdf]

S3 Table: Neutralization IC_{50} (µg/ml) and ID_{50} (1/dilution) titers are shown for ID_{50} (1/dilution) and IgG purified from sera from Day 359 for cow-485 and cow-488. Geomean IC_{50} and ID_{50} values are shown at the bottom of the graph.

Virus Strain	Clade	Bess1	Bess2	Bess4	ElsE1	ElsE2	Cow-485	Cow-488
6535.3	B	>50	>50	>50	>50	>50	<35	122
SC422661.8	B	>50	>50	>50	>50	>50	36	<35
PVO.4	B	>50	>50	>50	>50	>50	<35	<35
TRO.11	B	>50	>50	>50	>50	>50	<35	<35
AC10.0.29	B	>50	>50	>50	>50	>50	<35	<35
RHPA4259.7	B	>50	>50	>50	>50	>50	<35	<35
TRJO4551.58	B	>50	>50	>50	>50	>50	<35	<35
WITO4160.33	B	>50	>50	>50	>50	>50	<35	152
CAAN5342.A2	B	>50	>50	>50	>50	>50	<35	<35
WEAU_d15_410_5017	B (T/F)	>50	>50	>50	>50	>50	<35	<35
1006_11_C3_1601	B (T/F)	>50	>50	>50	>50	>50	<35	<35
1056_10_TA11_1826	B (T/F)	>50	>50	>50	>50	>50	<35	43
1012_11_TC21_3257	B (T/F)	>50	>50	>50	>50	>50	<35	56
6240_08_TA5_4622	B (T/F)	>50	>50	>50	>50	>50	<35	<35
6244_13_B5_4576	B (T/F)	>50	>50	>50	>50	>50	<35	<35
62357_14_D3_4589	B (T/F)	>50	>50	>50	>50	>50	<35	<35
Du156.12	C	0.016	0.084	0.006	0.004	0.001	120	11126
ZM197M.PB7	C	>50	20	>50	36	0.607	<35	114
ZM214M.PL15	C	>50	>50	>50	>50	>50	318	<35
ZM233M.PB6	C	>50	5	0.508	0.119	0.100	822	3830
ZM249M.PL1	C	0.014	0.029	0.015	0.450	0.032	92	318
ZM53M.PB12	C	>50	>50	0.014	10	0.049	98	280
ZM109F.PB4	C	>50	0.496	0.056	0.084	0.020	224	1485
ZM135M.PL10a	C	>50	>50	>50	>50	>50	<35	<35
CAP45.2.00.G3	C	0.001	0.002	0.0006	0.027	0.0006	2126	5804
CAP210.2.00.E8	C	>50	13	0.026	0.289	0.078	<35	154
HIV-0013095-2.11	C	>50	>50	0.023	0.043	0.007	38	6901
HIV-16055-2.3	C	0.001	0.014	0.0006	0.004	0.0006	1017	14435
Ce0393_C3	C (T/F)	>50	0.302	0.008	0.387	0.033	241	25670
Ce1176_A3	C (T/F)	>50	>50	0.049	8	0.004	68	672
Ce2010_F5	C (T/F)	>50	>50	>50	>50	>50	<35	<35
Ce0682_E4	C (T/F)	>50	>50	>50	>50	>50	<35	40
Ce1172_H1	C (T/F)	>50	>50	0.045	0.968	0.033	50	358
Ce2060_G9	C (T/F)	0.190	50	>50	>50	2	412	218
Ce703010054_2A2	C (T/F)	>50	>50	>50	>50	>50	<35	<35
246F C1G	C (T/F)	0.001	0.003	>50	>50	>50	<35	58
249M B10	C (T/F)	0.028	0.118	0.044	1	0.083	69	345
ZM247v1(Rev-)	C (T/F)	11	6	5	>50	>50	<35	39
7030102001E5(Rev-)	C (T/F)	>50	>50	>50	>50	>50	<35	<35
1394C9G1(Rev-)	C (T/F)	0.551	0.168	0.249	2	0.004	2034	1345
Ce704809221_1B3	C (T/F)	>50	>50	>50	>50	>50	<35	<35
CNE19	BC	>50	>50	>50	>50	0.147	72	932
CNE20 (CRF07_BC)	BC	>50	>50	0.319	>50	0.299	<35	135
CNE17	BC	0.517	0.517	0.016	0.085	0.017	140	851

CNE30	BC	>50	>50	>50	>50	>50	<35	<35
CNE52	BC	0.290	0.290	>50	0.937	0.020	<35	498
CNE53	BC	>50	>50	>50	>50	>50	<35	<35
CNE58	BC	0.458	1	0.087	4	0.044	494	904
MS208.A1	A	>50	>50	18	0.566	0.082	<35	582
Q23.17	A	1	0.163	0.006	36	2	48	<35
Q461.e2	A	0.025	0.172	0.075	1	0.105	<35	321
Q769.d22	A	>50	>50	0.001	1	0.078	112	277
Q259.d2.17	A	0.009	0.016	0.127	7	0.024	<35	606
0330.v4.c3	A	11	0.167	0.105	0.145	0.0006	<35	4512
0260.v5.c36	A	>50	0.373	0.091	39	18	<35	<35
191955_A11	A (T/F)	0.005	0.007	0.374	1	0.001	1773	780
T257-31	CRF02_AG	0.035	0.057	0.003	0.027	0.001	2845	84
263-8	CRF02_AG	0.025	0.335	0.040	0.186	0.024	506	<35
T250-4	CRF02_AG	0.0006	0.002	0.067	0.005	0.006	6756	10458
T251-18	CRF02_AG	>50	>50	>50	>50	>50	<35	<35
T278-50	CRF02_AG	>50	>50	>50	14	1	<35	<35
T255-34	CRF02_AG	>50	>50	>50	>50	>50	<35	<35
211-9	CRF02_AG	>50	>50	>50	>50	>50	<35	<35
235-47	CRF02_AG	>50	>50	>50	24	3	<35	<35
620345.c01	CRF01_AE	0.080	0.055	0.001	0.087	0.006	160	2238
C1080.c03	CRF01_AE	>50	5	0.014	0.0006	0.0006	15010	145536
R2184.c04	CRF01_AE	0.062	0.654	0.007	0.481	0.024	168	547
R1166.c01	CRF01_AE	>50	>50	>50	>50	>50	393	151
R3265.c06	CRF01_AE	>50	1	0.014	0.096	0.016	671	611
C3347.c11	CRF01_AE	12	0.545	>50	>50	>50	<35	<35
C4118.c09	CRF01_AE	0.005	0.021	0.0006	0.0006	0.0006	1403	11276
CNE8	CRF01_AE	0.386	0.057	0.263	0.029	0.005	581	4048
CNE5	CRF01_AE	0.001	0.010	0.184	0.027	0.0006	2300	5281
BJOX009000.02.4	CRF01_AE	>50	>50	>50	>50	>50	<35	58
BJOX015000.11.5	CRF01_AE (T/F)	>50	>50	>50	>50	>50	293	63
BJOX010000.06.2	CRF01_AE (T/F)	>50	>50	>50	>50	>50	<35	<35
BJOX025000.01.1	CRF01_AE (T/F)	>50	>50	2	>50	>50	268	53
X1193_C1	G	>50	>50	>50	>50	>50	<35	<35
P0402_C2_11	G	>50	>50	16	>50	26	<35	103
X1254_C3	G	>50	>50	>50	>50	>50	<35	<35
X2088_C9	G	>50	>50	0.017	0.087	0.022	456	2122
X2131_C1_B5	G	>50	>50	>50	>50	>50	<35	<35
P1981_C5_3	G	0.119	3	0.031	0.112	0.007	78	1945
X1632_S2_B10	G	11	>50	>50	0.231	0.024	169	461
3016.v5.c45	D	>50	26	>50	37	0.384	<35	35
A07412M1.vrc12	D	0.582	0.993	50	3	0.237	41	261
231965.c01	D	>50	>50	0.040	>50	0.088	<35	327
231966.c02	D	>50	>50	>50	>50	>50	<35	108
3817.v2.c59	CD	0.034	2	0.018	0.401	0.0006	197	7236
6480.v4.c25	CD	>50	>50	>50	>50	>50	<35	<35
6952.v1.c20	CD	>50	>50	>50	>50	>50	<35	<35
6811.v7.c18	CD	0.035	0.059	>50	>50	>50	<35	<35
89-F1_2_25	CD	>50	>50	>50	>50	7	<35	<35

3301.v1.c24	AC	0.245	>50	0.269	8	0.454		<35	45
6041.v3.c23	AC	0.018	0.086	>50	>50	>50		<35	<35
6545.v4.c1	AC	0.014	0.065	0.003	0.005	0.001		35	4006
0815.v3.c3	ACD	>50	>50	>50	>50	>50		<35	<35
3103.v3.c10	ACD	>50	>50	>50	>50	>50		<35	<35
	Geomean IC_{50}	0.061	0.238	0.047	0.352	0.031	Geomean ID_{50}	291	588

Neutralization IC_{50} (µg/ml)				
50	1	0.5	0.05	0.005

Neutralization ID_{50} (1/Dilution)				
35	150	500	1000	5000

S4 Table: Table of recovered heavy chains tested with native and universal light chains.

Sort Stats and Summary of Screening			Sort 1	Sort 2	Sort 3	Sort 4	Short CDRH3 (0-24 AA)	Long CDRH3 (25-49 AA)	Ultralong CDRH3 (50+ AA)
Cow 485	All	Cells Sorted	108	453	559	438	NA	NA	NA
		PCR Wells Positive for Heavy Chains	34	75	169	52	NA	NA	NA
		Heavy Chain Sequences Recovered	13	43	47	49	42	72	38
	Universal Light Chain	Tested IGHV-1*7 Heavy Chains	10	40	30	NT	0	52	28
		Expressed in Screen	9	22	25	NT	0	32	24
		Positive for BG505 Binding	8	13	12	NT	0	14	19
		Monoclonals with Cross-Clade Neutralization	1	1	4	2	0	0	8
	Native Light Chain	Heavy Chain/Light Chain Pairs	12	32	41	49	41	63	30
		Expressed in Screen	9	24	34	45	34	53	25
		Positive for BG505 Binding	7	12	12	20	13	22	16
		Monoclonals with Cross-Clade Neutralization	1	0	3	2	0	0	6
Cow 488	All	Cells Sorted	33	342	636	283	NA	NA	NA
		PCR Wells Positive for Heavy Chains	6	13	58	54	NA	NA	NA
		Heavy Chain Sequences Recovered	1	6	17	49	34	4	35
	Universal Light Chain	IGHV-1*7 Heavy Chains	0	2	1	NT	1	2	0
		Expressed in Screen	0	2	0	NT	0	2	0
		Positive for BG505 Binding	0	2	0	NT	0	2	0
		Monoclonals with Cross-Clade Neutralization	0	0	0	11	0	0	0
	Native Light Chains	Heavy Chain/Light Chain Pairs	1	5	16	49	33	3	35
		Expressed in Screen	1	5	10	44	25	3	32
		Positive for BG505 Binding	0	3	3	33	8	3	28
