# Supplementary material for: Immunization of cows with HIV envelope trimers generates broadly neutralizing antibodies to the V2-apex from the ultralong CDRH3 repertoire
Source: PLoS Pathog. 2024 Sep 9;20(9):e1012042. doi: 10.1371/journal.ppat.1012042 (PMC11412654; doi:10.1371/journal.ppat.1012042)
Supplement: S4 Table — (PDF) [file ppat.1012042.s016.pdf]

S4 Table: Table of recovered heavy chains tested with native and universal light chains.

Sort Stats and Summary of Screening			Sort 1	Sort 2	Sort 3	Sort 4	Short CDRH3 (0-24 AA)	Long CDRH3 (25-49 AA)	Ultralong CDRH3 (50+ AA)
Cow 485	All	Cells Sorted	108	453	559	438	NA	NA	NA
		PCR Wells Positive for Heavy Chains	34	75	169	52	NA	NA	NA
		Heavy Chain Sequences Recovered	13	43	47	49	42	72	38
	Universal Light Chain	Tested IGHV-1*7 Heavy Chains	10	40	30	NT	0	52	28
		Expressed in Screen	9	22	25	NT	0	32	24
		Positive for BG505 Binding	8	13	12	NT	0	14	19
		Monoclonals with Cross-Clade Neutralization	1	1	4	2	0	0	8
	Native Light Chain	Heavy Chain/Light Chain Pairs	12	32	41	49	41	63	30
		Expressed in Screen	9	24	34	45	34	53	25
		Positive for BG505 Binding	7	12	12	20	13	22	16
		Monoclonals with Cross-Clade Neutralization	1	0	3	2	0	0	6
Cow 488	All	Cells Sorted	33	342	636	283	NA	NA	NA
		PCR Wells Positive for Heavy Chains	6	13	58	54	NA	NA	NA
		Heavy Chain Sequences Recovered	1	6	17	49	34	4	35
	Universal Light Chain	IGHV-1*7 Heavy Chains	0	2	1	NT	1	2	0
		Expressed in Screen	0	2	0	NT	0	2	0
		Positive for BG505 Binding	0	2	0	NT	0	2	0
		Monoclonals with Cross-Clade Neutralization	0	0	0	11	0	0	0
	Native Light Chains	Heavy Chain/Light Chain Pairs	1	5	16	49	33	3	35
		Expressed in Screen	1	5	10	44	25	3	32
		Positive for BG505 Binding	0	3	3	33	8	3	28
