# Supplementary material for: Immunization of cows with HIV envelope trimers generates broadly neutralizing antibodies to the V2-apex from the ultralong CDRH3 repertoire
Source: PLoS Pathog. 2024 Sep 9;20(9):e1012042. doi: 10.1371/journal.ppat.1012042 (PMC11412654; doi:10.1371/journal.ppat.1012042)
Supplement: S5 Table — IC50 (μg/ml), IC80 (μg/ml), and MPN (%) are shown for all antibodies whose IC50 reach at least 50% neutralization. MPN = Maximum Percent Neutralized. (PDF) [file ppat.1012042.s017.pdf]

S5 Table: ElsE and Bess antibodies were tested for their ability to neutralize the 12-virus global panel. IC_{50} (µg/ml), IC_{80} (µg/ml), and MPN (%) are shown for all antibodies whose IC_{50} reach at least 50% neutralization. MPN= Maximum Percent Neutralized.

		398F1	TRO.11	X2278	25710	CE0217	Ce1176	X1632	246F3	CNE55	CNE8	BJOX	CH119
		A	B	B	C	C	C	G	AC	AE	AE	BC	BC
Bess1	IC_{50} (µg/ml)	>50	>50	>50	<.005	>50	>50	11	0.054	<.005	0.386	0.066	>50
	IC_{80} (µg/ml)	>50	>50	>50	0.013	>50	>50	>50	0.215	<.005	2	0.315	>50
	MPN	<50	<50	<50	100%	<50	<50	58%	94%	100%	90%	94%	<50
Bess2	IC_{50} (µg/ml)	>50	>50	>50	0.014	0.355	>50	>50	0.03	<.005	0.057	0.232	>50
	IC_{80} (µg/ml)	>50	>50	>50	0.057	1	>50	>50	0.121	0.019	0.229	0.928	>50
	MPN	<50	<50	<50	99%	88%	<50	<50	99%	100%	99%	98%	<50
Bess3	IC_{50} (µg/ml)	>50	>50	>50	0.067	>50	>50	6	0.063	0.042	0.603	0.067	>50
	IC_{80} (µg/ml)	>50	>50	>50	0.268	>50	>50	>50	0.250	0.170	2	0.270	>50
	MPN	<50	<50	<50	100%	<50	<50	62%	99%	100%	97%	99%	<50
Bess4	IC_{50} (µg/ml)	>50	>50	>50	0.064	0.127	0.049	>50	0.245	0.015	0.263	0.025	>50
	IC_{80} (µg/ml)	>50	>50	>50	0.258	0.509	0.195	>50	0.982	0.061	1	0.098	>50
	MPN	<50	<50	<50	98%	95%	93%	<50	92%	100%	100%	99%	<50
Bess5	IC_{50} (µg/ml)	>50	>50	>50	>50	24	13	>50	>50	0.02	0.093	14	>50
	IC_{80} (µg/ml)	>50	>50	>50	>50	>50	>50	>50	>50	0.079	1	>50	>50
	MPN	<50	<50	<50	<50	50%	56%	<50	<50	97%	91%	62%	<50
Bess6	IC_{50} (µg/ml)	>50	>50	49	41	>50	17	>50	>50	>50	>50	>50	>50
	IC_{80} (µg/ml)	>50	>50	>50	>50	>50	34	>50	>50	>50	>50	>50	>50
	MPN	<50	<50	52%	58%	<50	87%	<50	<50	<50	<50	<50	<50
Bess7	IC_{50} (µg/ml)	>50	>50	>50	0.477	>50	>50	>50	>50	<.005	>50	31	>50
	IC_{80} (µg/ml)	>50	>50	>50	<50	>50	>50	>50	>50	0.02	>50	<50	>50
	MPN	<50	<50	<50	80%	<50	<50	<50	<50	98%	<50	50%	<50
Bess8	IC_{50} (µg/ml)	>50	>50	>50	0.009	>50	>50	>50	2	0.007	9	0.21	>50
	IC_{80} (µg/ml)	>50	>50	>50	0.525	>50	>50	>50	7	0.029	>50	0.839	>50
	MPN	<50	<50	<50	97%	<50	<50	<50	72%	97%	59%	84%	<50
ElsE1	IC_{50} (µg/ml)	>50	>50	>50	<.005	2	8	0.231	0.058	<.005	0.029	0.033	>50
	IC_{80} (µg/ml)	>50	>50	>50	0.019	>50	>50	0.924	0.233	0.019	0.116	0.133	>50
	MPN	<50	<50	<50	99%	74%	66%	81%	88%	99%	100%	100%	<50
ElsE2	IC_{50} (µg/ml)	>50	>50	>50	0.006	0.009	<.005	0.024	0.007	<.005	0.005	0.023	>50
	IC_{80} (µg/ml)	>50	>50	>50	0.023	0.037	0.018	0.096	0.027	0.014	0.022	0.091	>50
	MPN	<50	<50	<50	100%	94%	94%	84%	96%	99%	100%	100%	<50
ElsE3	IC_{50} (µg/ml)	>50	>50	>50	<.005	0.009	0.062	0.161	0.153	0.007	0.009	0.026	>50
	IC_{80} (µg/ml)	>50	>50	>50	0.015	0.035	0.249	0.643	0.611	0.027	0.034	0.103	>50
	MPN	<50	<50	<50	100%	97%	92%	84%	90%	98%	100%	100%	<50
ElsE4	IC_{50} (µg/ml)	>50	>50	>50	0.016	23	>50	1	2	<.005	0.011	0.035	>50
	IC_{80} (µg/ml)	>50	>50	>50	0.062	>50	>50	>50	>50	0.012	0.042	0.139	>50
	MPN	<50	<50	<50	98%	54%	<50	74%	78%	98%	100%	100%	<50
ElsE5	IC_{50} (µg/ml)	>50	>50	>50	0.008	26	>50	3	3	0.008	0.011	0.031	>50
	IC_{80} (µg/ml)	>50	>50	>50	0.032	>50	>50	>50	>50	0.031	0.044	0.123	>50
	MPN	<50	<50	<50	99%	52%	<50	68%	74%	98%	100%	99%	<50
ElsE6	IC_{50} (µg/ml)	>50	>50	>50	0.06	>50	>50	5	>50	0.033	0.023	0.013	>50
	IC_{80} (µg/ml)	>50	>50	>50	0.238	>50	>50	>50	>50	0.13	0.093	0.051	>50
	MPN	<50	<50	<50	91%	<50	<50	68%	<50	95%	99%	99	<50
ElsE7	IC_{50} (µg/ml)	>50	>50	>50	0.007	0.770	5	0.022	2	<.005	0.007	0.019	>50
	IC_{80} (µg/ml)	>50	>50	>50	0.028	3	>50	0.087	>50	0.019	0.026	0.076	>50
	MPN	<50	<50	<50	99%	80%	68%	91%	78%	99%	99%	100%	<50
ElsE8	IC_{50} (µg/ml)	>50	>50	>50	0.023	3	10	0.654	0.056	0.024	0.023	0.067	>50
	IC_{80} (µg/ml)	>50	>50	>50	0.094	>50	>50	>50	0.223	0.096	0.092	0.267	>50
	MPN	<50	<50	<50	99%	71%	60%	74%	90%	97%	99%	100%	<50
ElsE9	IC_{50} (µg/ml)	>50	>50	>50	0.017	0.723	3	0.067	0.047	0.006	0.019	0.043	>50
	IC_{80} (µg/ml)	>50	>50	>50	0.067	>50	>50	0.269	0.188	0.022	0.077	0.172	>50
	MPN	<50	<50	<50	99%	75%	69%	81%	92%	99%	100%	100%	<50
ElsE10	IC_{50} (µg/ml)	>50	>50	>50	0.025	>50	17	11	>50	0.009	0.063	0.093	>50
	IC_{80} (µg/ml)	>50	>50	>50	0.102	>50	>50	>50	>50	0.12	0.167	0.372	>50
	MPN	<50	<50	<50	93%	<50	60%	62%	<50	96%	98%	93%	<50
ElsE11	IC_{50} (µg/ml)	>50	>50	>50	0.033	>50	>50	0.772	>50	0.016	0.031	0.027	>50
	IC_{80} (µg/ml)	>50	>50	>50	0.134	>50	>50	>50	>50	0.064	0.161	0.283	>50
	MPN	<50	<50	<50	93%	<50	<50	72%	<50	96%	98%	99%	<50
