# Supplementary material for: Immunization of cows with HIV envelope trimers generates broadly neutralizing antibodies to the V2-apex from the ultralong CDRH3 repertoire
Source: PLoS Pathog. 2024 Sep 9;20(9):e1012042. doi: 10.1371/journal.ppat.1012042 (PMC11412654; doi:10.1371/journal.ppat.1012042)
Supplement: S6 Table — (PDF) [file ppat.1012042.s018.pdf]

S6 Table: X-ray data collection and refinement statistics for Bess and ElsE Fabs.

	Fab ElsE1	Fab ElsE2	Fab ElsE5	Fab ElsE6	Fab ElsE7	Fab ElsE8	Fab ElsE9	Fab ElsE11	Fab Bess4	Fab Bess7
**PDB code**	8V4I	8VBJ	8VBK	8VBL	8VBM	8VBN	8VBO	8VBR	8VBP	8VBQ
**Crystallization condition**	40% ethylene glycol, 0.1M HEPES, pH 7.5, 5% Peg3000, 20°	0.1M Tris, pH 8.5, 1.5M ammonium sulfate, 12% glycerol, 4°	0.1M sodium cacodylate, pH 6.5, 40% MPD, 5% Peg8000, 20°	0.1M Ches, pH 9.5, 50% Peg200, 20°	0.1M Ches, pH 9.5, 20% Peg 8000, 4°	20% Peg3350, 0.2M KCl, 4°	0.1M sodium cacodylate, pH 6.5, 40% MPD, 5% Peg 8000, 20°	0.1M Ches, pH 9.5, 50% Peg200, 4°	70% MPD, 0.1M Hepes, pH 7.5, 20°	0.095M sodium citrate, pH 5.6, 19% 2-propanol, 5% glycerol, 19% Peg4000, 20°
**Wavelength**	0.97946	0.97946	1.03317	0.97946	0.97946	0.97741	0.97946	0.97741	0.97946	0.97741
**Beamline**	SSRL 12-1	SSRL 12-2	APS 23-ID-B	SSRL 12-2	SSRL 12-2	ALS 5.0.1	SSRL 12-1	ALS 5.0.1	SSRL 12-1	ALS 5.0.1
**Resolution range**	37.89 - 1.81 (1.88 - 1.81)	45.48 - 1.90 (1.97 - 1.90)	43.86 - 1.89 (1.96 - 1.89)	46.80 - 2.35 (2.43 - 2.35)	42.23 - 2.54 (2.63 - 2.54)	46.95 - 1.83 (1.90 - 1.83)	36.30 - 2.30 (2.38 - 2.30)	49.58 - 2.65 (2.75 - 2.65)	29.94 - 2.80 (2.90 - 2.80)	48.29 - 2.10 (2.18 - 2.10)
**Space group/twin law**	C 2 2 2_1	C 2 2 2_1	P $2_1 2_1 2_1$	P 2_1 2_1 2	P 2_1 2_1 2_1	P 2_1 2_1 2_1	P 2_1 2_1 2	P 2_1 2_1 2_1 l,h,-k	P 2_1 2_1 2_1	C 2
**Unit cell (Å,°)**	69.79, 84.12, 174.58, 90, 90, 90	69.96, 178.59, 105.71, 90, 90, 90	56.26, 70.02, 109.08, 90, 90, 90	117.59, 69.98, 77.32, 90, 90, 90	58.22, 70.61, 123.97, 90, 90, 90	59.22, 120.27, 150.30, 90, 90, 90	70.11, 150.89, 51.32, 90, 90, 90	70.05, 70.19, 241.83, 90, 90, 90	66.61, 70.28, 111.46, 90, 90, 90	83.00, 69.78, 87.52, 90, 103.52, 90
**Total reflections**	460,645 (32,773)	442,490 (44,977)	423,944 (39,845)	168,777 (17,085)	76,645 (6407)	1,219,769 (90,327)	81,165 (7982)	378,268 (10,647)	44,117 (4130)	98,588 (7823)
**Unique reflections**	45,028 (3292)	51,939 (5160)	34,653 (3286)	27,185 (2649)	16,971 (1511)	95,768 (9306)	24,379 (2325)	33,457 (1618)	12,710 (1143)	28,113 (2607)
**Multiplicity**	10.2 (10.0)	8.5 (8.7)	12.2 (12.1)	6.2 (6.4)	4.5 (4.2)	12.8 (9.8)	3.3 (3.4)	11.3 (6.6)	3.5 (3.3)	3.5 (3.0)
**Completeness (%)**	95.6 (70.1)	98.8 (99.2)	98.7 (94.9)	99.7 (98.8)	96.8 (84.6)	99.8 (98.2)	97.8 (95.3)	94.0 (63.9)	93.8 (87.2)	98.8 (92.4)
**Mean I/sigma(I)**	10.5 (0.9)	8.4 (1.4)	13.8 (2.4)	9.1 (2.4)	7.5 (1.5)	15.9 (1.3)	9.3 (2.5)	11.3 (6.6)	7.0 (3.0)	8.7 (1.2)
**Wilson B-factor**	34	23	27	34	44	26	44	51	38	32
**R-merge (%)**	15.5 (251)	22.4 (224)	11.8 (109)	19.8 (140)	16.4 (135)	11.6 (173)	9.2 (77.8)	9.0 (84.8)	19.1 (80.5)	15.2 (107)
**R-meas (%)**	16.2 (264)	23.8 (238)	12.3 (114)	21.7 (152)	18.6 (155)	12.0 (183)	10.8 (90.9)	9.4 (91.4)	22.4 (95.9)	17.9 (128)
**R-pim (%)**	4.6 (77.2)	7.9 (77.6)	3.5 (32.3)	8.6 (59.4)	8.6 (74.2)	3.3 (57.5)	5.6 (46.2)	2.7 (32.0)	11.5 (51.0)	9.3 (70.3)
**CC1/2 (%)**	99.6 (41.0)	99.5 (41.3)	99.8 (86.7)	99.3 (49.7)	99.0 (40.0)	99.9 (50.8)	99.5 (61.1)	99.0 (77.4)	97.3 (49.8)	99.2 (37.6)
**Reflections used in refinement**	44,983 (3264)	51,917 (5148)	34,637 (3277)	27,183 (2649)	16,886 (1437)	95,655 (9237)	24,379 (2325)	33,316 (2271)	12,577 (1141)	28,099 (2601)
**Reflections used for R-free**	2273 (190)	2580 (266)	1773 (159)	1331 (141)	783 (77)	1970 (191)	1175 (117)	1993 (138)	620 (62)	1348 (114)
**R-work (%)**	22.1 (41.1)	19.5 (35.0)	20.4 (33.9)	19.7 (27.7)	23.5 (37.8)	22.4 (37.5)	24.8 (34.1)	24.7 (49.7)	19.6 (26.2)	23.0 (32.5)
**R-free (%)**	25.7 (55.6)	22.9 (37.2)	24.5 (39.3)	24.4 (34.5)	29.0 (46.8)	25.8 (39.6)	28.0 (38.9)	26.0 (56.4)	23.2 (30.9)	26.7 (30.7)
**Number of non-hydrogen atoms**	3750	3889	3707	3593	3463	7146	3535	7026	3257	3715
**macromolecules**	3563	3555	3496	3477	3463	6755	3465	7026	3257	3569
**solvent**	191	334	211	116	0	681	70	0	0	142
**Protein residues**	479	477	473	475	472	915	469	956	441	486
**RMS(bonds)**	0.003	0.013	0.003	0.002	0.002	0.008	0.002	0.003	0.003	0.003

**RMS(angles)**	0.66	1.05	0.64	0.56	0.51	0.98	0.53	0.56	0.58	0.56
**Ramachandran favored, allowed, outliers (%)**	94.3, 5.3, 0.4	97.5, 2.5, 0.0	96.8, 3.2, 0.0	96.2,3.4, 0.4	94.6, 4.7, 0.6	96.56, 3.33, 0.11	94.58, 4.34, 1.08	95.25, 4.11, 0.63	95.17, 4.37, 0.46	95.23, 4.77, 0.00
**Rotamer outliers (%)**	1.94	2.18	0.99	1.23	1.75	1.79	1.76	1.84	1.58	0.73
**Clashscore**	3.3	1.9	1.3	3.2	4.6	2.4	3.7	2.9	2.2	1.7
**Average B-factor**	45	31	35	43	65	35	63	59	36	47
**macromolecules**	45	31	35	43	65	35	63	59	36	48
**solvent**	44	35	36	41	NA	37	72	NA	NA	37

Statistics for the highest-resolution shell are shown in parentheses.
