# Supplementary material for: Immunization of cows with HIV envelope trimers generates broadly neutralizing antibodies to the V2-apex from the ultralong CDRH3 repertoire
Source: PLoS Pathog. 2024 Sep 9;20(9):e1012042. doi: 10.1371/journal.ppat.1012042 (PMC11412654; doi:10.1371/journal.ppat.1012042)
Supplement: S7 Table — # = residue number, AA = residue type, and SS = secondary structure type. DSSP was used to assign the secondary structure type for residues in stalk/knob regions from the crystal structures [86]. SS are designated as: α-helix: H, 310 helix: G, extended strand: E, bend: S, hydrogen bonded turn: T. Blank fields have ‘coil’ secondary structure. Blank AA fields indicate unmodeled residues. Residues at the tips of the two knob ‘loops’ (114–118 and 127–133 in the ElsE Fabs and 114–120 and 132–137 in Bess7) are highlighted in bold; these might be expected to interact with Env. (PDF) [file ppat.1012042.s019.pdf]

S7 Table: Secondary structural elements in stalk/knob regions. #=residue number, AA=residue type, and SS=secondary structure type. DSSP was used to assign the secondary structure type for residues in stalk/knob regions from the crystal structures [86]. SS are designated as: α-helix: H, 3_{10} helix :G, extended strand: E, bend: S, hydrogen bonded turn: T. Blank fields have 'coil' secondary structure. Blank AA fields indicate unmodeled residues. Residues at the tips of the two knob 'loops' (114-118 and 127 -133 in the ElsE Fabs and 114-120 and 132-137 in Bess7) are highlighted in bold; these might be expected to interact with Env.

	Bess7		ElsE1		ElsE2		ElsE5		ElsE6		ElsE7		ElsE8		ElsE9		ElsE11_1		ElsE11_2	
**#**	**AA**	**SS**	**AA**	**SS**	**AA**	**SS**	**AA**	**SS**	**AA**	**SS**	**AA**	**SS**	**AA**	**SS**	**AA**	**SS**	**AA**	**SS**	**AA**	**SS**
**92**	C		C		C		C		C		C		C		C		C		C	
**93**	T		T		T		T		T		T		T		T		T		T	
**94**	T	E	T	E	T	E	T	E	T	E	T	E	A	E	T		T	E	T	
**95**	V	E	V	E	V	E	V	E	V	E	V	E	V	E	V	E	V	E	V	E
**96**	H	E	H	E	H	E	H	E	H	E	H	E	V	E	R	E	R	E	R	E
**97**	Q	E	Q	E	Q	E	Q	E	Q	E	Q	E	Q	E	Q	E	Q	E	Q	E
**98**	T	E	Q	E	Q	E	Q	E	Q	E	Q	E	E	E	Q	E	Q	E	Q	E
**99**	T	E	T	E	T	E	T	E	T	E	T	E	T	E	V	E	T	E	T	E
**100**	K	E	R	E	R	E	R	E	R	E	R	E	R	E	H	E	R	E	R	E
**101**	T		K	E	K	E	K	E	K	E	K	E	K	E	K	E	K	E	K	E
**102**	T		G	E	S	E	G	E	S	E	S	E	S	E	T	E	S	E	S	E
**103**	K	E	C		C		C		C		C		C		C		C		C	
**104**	E	E	P		P		P		P		P		P		P		P		P	
**105**	C		D	T	G	T	D	S	A	S	D	S	D	T	Q	S	D	T	D	T
**106**	P		G	T	G	T	G	S	G	S	G	S	G	T	G	S	G	T	G	T
**107**	E	T	W		Y		W		Y		W		W		W		W		W	
**108**	G	T	R	E	T	E	S	E	T	E	M	E	R	E	R	E	T	E	T	E
**109**	Y		F	E	F	E	F	E	L	E	F	E	F	E	F	E	L	E	L	E
**110**	N	E	G	H	G	G	G	T	A	T	G	S	G	T	G	H	A	H	A	H
**111**	W	E	W	H	Y	G	W	T	K	T	F	T	W	T	W	H	K	H	K	H
**112**	D		D	H	D	G	D	T	D	T	D	T	D	T	D	H	D	H	D	H
**113**	D	S	C	H	C	G	C	T	C	T	C		C	T	C	H	C	H	C	H
**114**	**G**		**G**	**T**	**G**	**T**	**G**	**T**	**G**				**G**	**T**	**G**	**T**	**G**	**T**	**G**	**T**
**115**	**C**		**F**		**F**		**F**						**F**		**F**		**F**		**F**	
**116**	**G**		**H**		**H**								**H**		**H**		**Y**		**Y**	
**117**	**S**	**G**	**G**	**S**	**G**	**S**							**G**	**S**	**G**	**S**	**G**	**S**	**G**	**S**
**118**	**E**	**G**	**Y**	**T**	**W**	**T**							**Y**	**T**	**Y**	**T**	**Y**	**T**	**Y**	**T**
**119**	**L**	**G**	G	T	G	T			G		G		G	T	G	T	G	T	G	T
**120**	**G**		Q	T	S	T	!		S	T	S		T	T	S	T	S	T	S	T
**121**	C		E	T	D	T	E		E	T	E	S	E	T	D	T	E	T	E	T
**122**	G	S	D	S	D	S	D		D	S	D	S	D	S	D	S	D		D	S
**123**	G	S	C	E	C	E	C	E	C	E	C	E	C	E	C	E	C	E	C	E
**124**	A	S	Y	E	Y	E	Y	E	Y	E	V	E	Y	E	Y	E	Y	E	Y	E
**125**	D	S	E	S	P	S	D	S	D	S	D	S	E	S	E	S	D	S	D	S
**126**	C	S	D		D	S	D		D		D		D		D	S	D		D	

127	C		C	S	C	S	C	S	C	T	C	T	C	S	C		C	T	C	T
128	C	E	I	S	S	S	T	S	S	T	S	T	V	S	I	S	T	T	T	T
129	W	E	D	S	D		D		D	T	D	T	D		D	T	D		D	
130	G	E	I	G	I	T	I	T	I	S	I	S	I	T	I	T	I	T	I	S
131	G	E	L	G	L	T	L	T	L	S	L	S	L	T	L	S	L	T	L	T
132	V	E	S	G	N		S	S	S	S	S	S	S	T	S	S	S	T	S	T
133	D	E	S	S	S	T	S		S		A	S	S		S	S	S	S	S	
134	E	T	Q		D	T	Q		H		Q		E		Q		N		N	
135	Y	T	T	E	V		T		T	E	T	E	T	E	T	E	T	E	T	E
136	A	T	L	E	V	B	L	B	L	E	L	E	V	E	L	E	L	E	L	E
137	G	E	S		G		S		S		S		S		S		S		S	
138	D	E	P		P		P		P		P		S		A		P		P	
139	L	E	T	E	I	E	T	E	T	E	I	E	T	E	E	E	T	E	T	E
140	Y	E	D	E	D	E	D	E	T	E	Y	E	D	E	D	E	T	E	T	E
141	S	E	A	E	T	E	T	E	T	E	T	E	R	E	T	E	T	E	T	E
142	C		Y	E	H	E	Y	E	Y	E	N	E	Y	E	Y	E	H	E	H	E
143	C		E	E	E	E	E	E	E	E	A	E	E	E	E	E	E	E	E	E
144	S	E	F	E	F	E	F	E	L	E	Y	E	L	E	L	E	F	E	F	E
145	V	E	H	E	H	E	H	E	H	E	H	E	H	E	H	E	N	E	N	E
146	A		V	E	V	E	V	E	V	E	V	E	V	E	V	E	V	E	V	E
147	H		D	E	D	E	D	E	D	E	D	E	D	E	D		D	E	D	
148	T	E	A	E	A	E	A	E	A	E	A	E	A	E	A		A	E	A	
149	Y	E	W		W		W		W		W		W		W		W		W	
150	E	E																		
151	W	E																		
152	Y	E																		
153	V	E																		
154	D	E																		
155	A	E																		
156	W																			
