# Supplementary material for: Immunization of cows with HIV envelope trimers generates broadly neutralizing antibodies to the V2-apex from the ultralong CDRH3 repertoire
Source: PLoS Pathog. 2024 Sep 9;20(9):e1012042. doi: 10.1371/journal.ppat.1012042 (PMC11412654; doi:10.1371/journal.ppat.1012042)
Supplement: S8 Table — (PDF) [file ppat.1012042.s020.pdf]

S8 Table: Cryo-EM data collection, refinement and validation statistics.

	BG505 SOSIP + Bess4 Fab + RM20A3 Fab (EMD-41498) (PDB: 8TQ1)
**Data collection and processing**	
Microscope	TFS Krios
Magnification	130,000x
Voltage (kV)	300
Electron exposure (e–/Å^2)	55.5
Defocus range (μm)	-0.7 to -1.7
Detector	Gatan K2 Summit
Recording mode	Counting
Pixel size (Å)	1.045
Symmetry imposed	C1
Micrographs (no.)	3,871
Initial particle images (no.)	494,075
Final particle images (no.)	82,374
Map resolution (Å)<br>FSC threshold	3.3<br>0.143
Map sharpening *B* factor (Å^2)	-83.5
Map pixel size (Å)	1.045
Map resolution range (Å)	2.4-5.0
**Refinement**	
Initial model used (PDB code)	6x9r
Model resolution (Å)<br>FSC threshold	3.3<br>0.5
Model resolution range (Å)	2.4-4.0
EMRinger score	3.95
Model composition<br>Non-hydrogen atoms<br>Protein residues<br>Ligands	 19,788<br>2,400<br>79
Mean *B* factors (Å^2)<br>Protein<br>Ligand	 71<br>96
R.m.s. deviations<br>Bond lengths (Å)<br>Bond angles (°)	 0.006<br>1.125
Validation<br>MolProbity score<br>Clashscore<br>Poor rotamers (%)	 1.11<br>1.49<br>0.19
Ramachandran plot<br>Favored (%)<br>Allowed (%)<br>Disallowed (%)	 96.54<br>3.46<br>0.00
